# Identification of synthetic chemosensitivity genes paired with *BRAF* for *BRAF*/MAPK inhibitors

**DOI:** 10.1038/s41598-020-76909-2

**Published:** 2020-11-17

**Authors:** Kye Hwa Lee, Jinmin Goh, Yi-Jun Kim, Kwangsoo Kim

**Affiliations:** 1 Department of Biomedical Informatics, Asan Medical Center, Seoul, 05505 South Korea; 2 Biomedical Research Institute, Seoul National University Hospital, Seoul, 03080 South Korea; 3 Department of Chemical Engineering, Pohang University of Science and Technology (POSTECH), Pohang, 37673 South Korea; 4 Transdisciplinary Department of Medicine & Advanced Technology, Seoul National University Hospital, Seoul, 03080 South Korea; 5 Institute of Convergence Medicine, Ewha Womans University Mokdong Hospital, Seoul, 07985 South Korea

**Keywords:** Cancer therapeutic resistance, Targeted therapies, Cancer genomics, Synthetic biology, High-throughput screening

## Abstract

Molecular-targeted approaches are important for personalised cancer treatment, which requires knowledge regarding drug target specificity. Here, we used the synthetic lethality concept to identify candidate gene pairs with synergistic effects on drug responses. A synergistic chemo-sensitivity response was identified if a drug had a significantly lower half-maximal inhibitory concentration (IC_50_) in cell lines with a pair of mutated genes compared with those in other cell lines (wild-type or one mutated gene). Among significantly damaging mutations in the Genomics of Drug Sensitivity in Cancer database, we found 580 candidate synergistic chemo-sensitivity interaction sets for 456 genes and 54 commercial drugs. Clustering analyses according to drug/gene and drug/tissue interactions showed that BRAF/MAPK inhibitors clustered together; 11 partner genes for *BRAF* were identified. The combined effects of these partners on IC_50_ values were significant for both drug-specific and drug-combined comparisons. Survival analysis using The Cancer Genome Atlas data showed that patients who had mutated gene pairs in synergistic interaction sets had longer overall survival compared with that in patients with other mutation profiles. Overall, this analysis demonstrated that synergistic drug-responsive gene pairs could be successfully used as predictive markers of drug sensitivity and patient survival, offering new targets for personalised medicine.

## Introduction

Recent advances in molecular profiling techniques have contributed to obtaining unprecedented knowledge regarding the underlying pathogenic mechanisms of cancer development and progression, leading to more effective and rational therapeutic options, such as targeted therapy and immunotherapy^1^. Moreover, this shift has led to changes in standard clinical practice protocols for cancer diagnosis and treatment, including mandatory requirements for tests of a patient’s genomic signature to improve personalised therapy^2^. However, current actionable mutation targets are limited, despite continuous accumulation of cancer-specific genomic profiles^3^. The major bottleneck for the development of targeted anticancer drugs is related to the identification of drugs that can specifically target a patient’s cancer cell-specific molecular signature with minimal harm or off-target effects^4^. One approach to achieve such specificity is related to the concept of synthetic lethality (SL), a promising therapeutic strategy to selectively kill cancer cells based on the idea that two genes show an SL interaction if inactivation of one gene is compatible with viability, whereas inactivation of both leads to cell death^5^. Despite a long history of research on this concept, the identification of clinically actionable targets from SL interactions remains challenging because such phenomena are scarcely observed in vivo; indeed, cells with damaging gene pairs in an SL-type interaction will not be viable^6^. Furthermore, such experiments require the production of massive small interfering RNA libraries and testing for validation of the identified candidate SL pairs.

Based on the SL concept, a conditional SL interaction, or synthetic cytotoxicity (SC), is defined as a pair of dysfunctional genes that do not necessarily cause cell death but rather increase sensitivity to low, sublethal concentrations of a DNA-damaging agent^5^. Li et al.^7^ revealed that digenic interactions between *TEL1* and *ATM* mutations resulted in increased sensitivity to low-dose camptothecin in yeast. Such SC interactions have advantages compared with SL interactions because they can be directly observed in natural conditions. That is, when a pair of genes exhibits an SC interaction, the cells will be viable, even when mutations in the two genes occur simultaneously; however, the cells will be more sensitive to specific chemotherapy or changes in the surrounding cellular microenvironment^6,8^. Thus, this concept can be particularly useful in the design of chemotherapy because large numbers of cells with both mutations can be practically observed, and the viability of these cells in response to drug reactions can be easily tested by considering pairs of genes instead of single genes as markers to monitor treatment responses. Despite the attractiveness and theoretical potential of the SC interaction concept, this approach has rarely been applied or evaluated in practice in human subjects, the targets discussed to date have thus far been restricted to cytotoxic drugs^7^, or the analyses have focused on specific candidate genes^9^. Thus, if SC interactions are proven to be a general phenomenon, in which different gene pairs exhibit synergistic therapeutic responses to various types of anticancer therapies, multiple genes could be evaluated as candidate cancer therapy targets.

In the current study, we aimed to elucidate actionable synergistic chemotherapeutic responsiveness biomarkers. We first explored the chemotherapy sensitivity profiles of data from a public cancer cell line database to screen out groups of cell lines with mutated gene pairs and compare their drug sensitivity responses to those of other cell lines. To distinguish groups of genes that were mutually related to responses to drugs in the same class, we performed a clustering analysis using the significant gene pairs identified from our previous comparison and attempted to replicate the synergistic responses with survival data from a large cancer cohort. Finally, we explored the mutational profiles of genes reported to be related to the resistance mechanism of the screened out drug classes to expand the overall profiles of cancer-associated genes and candidate drug targets.

## Results

### Identification of candidate synergistic chemo-sensitivity (SCS) sets in cancer cell lines

Using the Genomics of Drug Sensitivity in Cancer (GDSC) database, we identified candidate SCS sets (gene pairs/drug) based on 7,233,306 comparisons for all gene pairs. The basic data (mean, standard deviation [SD], minimum and maximum values of gene pairs, genes, and cell lines per drug) of the final sets are provided in Supplementary Table S1, and the detailed information is shown in Supplementary Table S2. Several gene pairs, genes, and cell lines in each set showed substantial variations according to the drug. After filtering, 1580 significant SCS sets were identified for 157 drugs. After excluding noncommercial drugs, 580 final SCS interaction sets, including 456 genes for 54 commercial drugs, were obtained. The summary statistics of synergistic response gene pairs for the 54 drugs are represented in Supplementary Table S3.

### Candidate SCS sets identified by clustering analysis according to the tissue of origin

**Figure 1:**
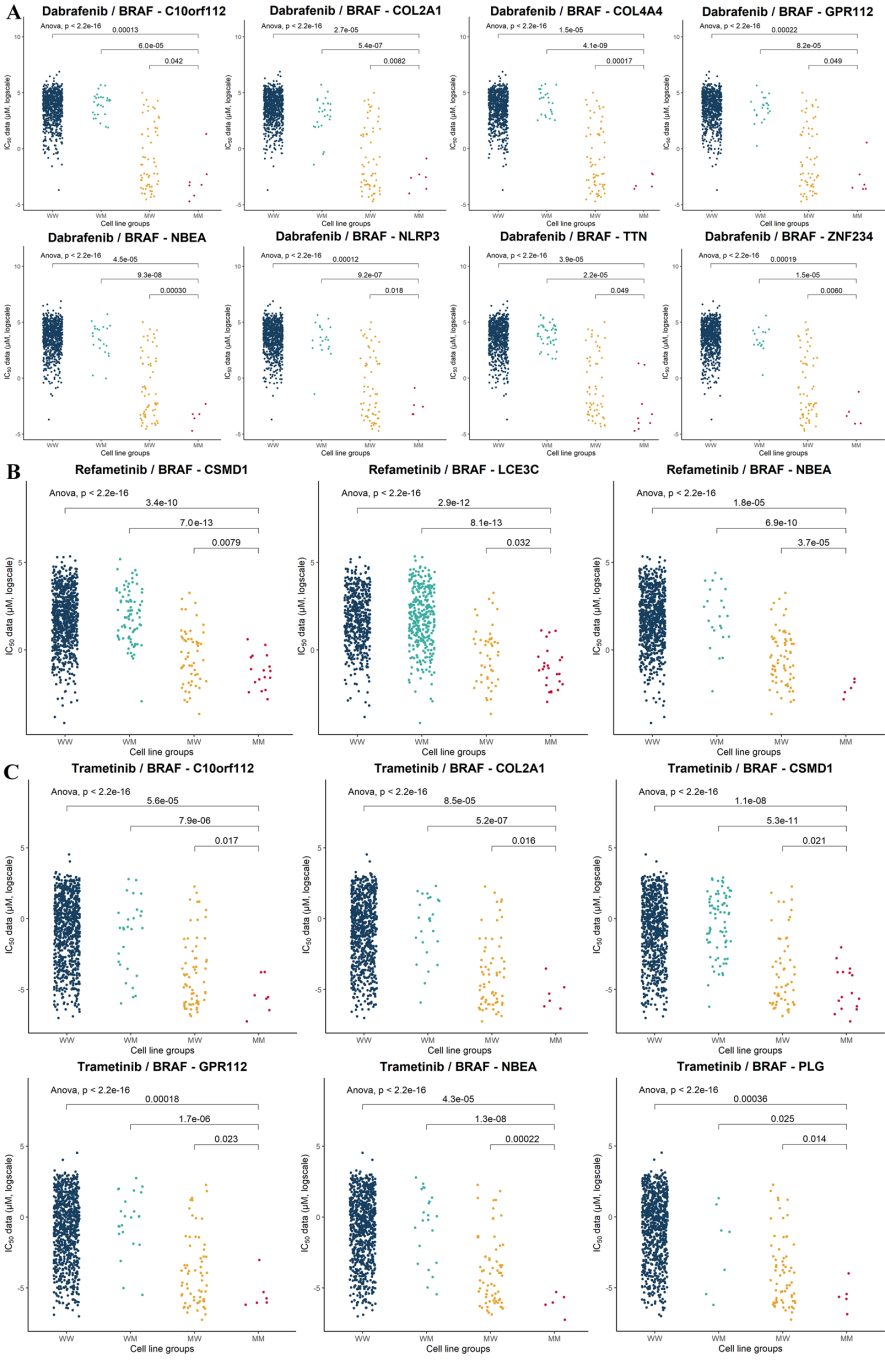
Comparison of IC_50_ values of four groups of cell lines according to the mutational profile of the synergistic interaction sets related to *BRAF* and BRAF/MAPK pathway-targeting drugs. Comparison of IC_50_ values for eight gene pairs according to the mutation profile of *BRAF* and partner genes for (**A**) dabrafenib, (**B**) refametinib, and (**C**) trametinib. W represents the wild-type gene, and M represents a mutated gene; WM represents the wild-type *BRAF* and mutated partner gene, and MW represents the mutated *BRAF* and wild-type partner gene in the SCS interaction pair. The comparison was performed by Student’s *t* test for the MM group and the other three groups and by ANOVA for comparisons of all groups.

These candidate SCS interaction sets contained a wide variety of genes and drugs (average of 10.73 gene pairs per drug for 54 drugs). Clustering analyses by drug/gene and drug/tissue pairs were performed to identify any common pathways or shared anatomical sites among these 54 drugs and 456 genes. For drug/gene clustering, only including genes with two or more damaging mutations (Supplementary Figure S1), three drugs classified as extracellular signal-regulated kinase (ERK)/mitogen-activated protein kinase (MAPK) inhibitors (dabrafenib, refametinib, and trametinib) clustered together based on shared *BRAF*-related synergistic interactions (Fig. 1). Although the GDSC database classifies these drugs as ERK/MAPK inhibitors, in this study, we used the more common classification of BRAF/MAPK inhibitors. Eighteen genes, including *BRAF*, among 14 gene pairs showed significant synergistic interactions with these three drugs. Table 1 lists the SCS interaction gene pairs identified for these three drugs and the frequency of cell lines with MM (mutated *BRAF* and partner genes) types for these pairs. As expected, *BRAF-*mutated cell lines showed significantly lower IC_50_ values than those of the WW (both *BRAF* and partner genes wild-type) or WM (wild-type *BRAF* and mutated partner genes) groups (Fig. 1). However, the MM (both genes mutated) cell lines had significantly lower IC_50_ values than the MW (mutated *BRAF* and wild-type partner genes) groups. Among the 861 cell lines with IC_50_ data available for dabrafenib, there were 66 *BRAF*-mutated cell lines (7.67%). Similarly, 72 (7.72%) of 933 cell lines and 70 (7.94%) of 882 cell lines harboured *BRAF* mutations for the refametinib and trametinib data, respectively. Among the nine gene pairs identified for dabrafenib, eight genes (*C10orf112*, *COL2A1*, *COL3A4*, *GPR112*, *NBEA*, *NLRP3*, *TTN*, and *ZNF234*) showed synergistic interactions with *BRAF*. Three of the four pairs identified for refametinib included *BRAF* with *CSMD1*, *LCE3C*, and *NBEA*, and six of the eight pairs for trametinib were related to *BRAF* with *C10orf112*, *COL2A1*, *CSMD1*, *GPR112*, *NBEA*, *NLRP3*, and *PLG* as partners. Thus, *NBEA* was a common partner gene of *BRAF* for all three drugs; *C10orf112*, *COL2A1*, and *GPR112* were common partners in the response to dabrafenib and trametinib; *CSMD1I* appeared for refametinib and trametinib; and *LCE3C*, *PLG*, *TTN*, and *ZNF234* appeared only once for a specific drug. In total, there were 28, 37, and 28 cell lines including MM groups for *BRAF* and its partners for dabrafenib, refametinib, and trametinib, respectively. There were also three pairs of genes with non-*BRAF*-related SCS interaction sets for these three drugs, among which *ABCA12-PDZD2* and *C7-USH2A* shared some of the same MM cell lines with the *BRAF*-related gene pairs. Otherwise, the pair *LRRTM3-SCN1A* occurred in novel MM cell lines that did not overlap with those harbouring *BRAF*-related gene pairs.

**Table 1:** Numbers of cell lines having mutations in both *BRAF* and partner genes grouped by the three BRAF/MAPK inhibitors**.**

	Gene pairs in the SCS sets	Number of MM-positive cell lines*		
		Dabrafenib (n = 861)	Refametinib (n = 933)	Trametinib (n = 882)
*BRAF-*related gene pairs	Number of cell lines with MM types in *BRAF*-related sets	n = 66	n = 72	n = 70
	*BRAF–C10orf112*	7 (10.61%)	–	7 (10.00%)
	*BRAF–COL2A1*	6 (9.09%)	–	6 (8.57%)
	*BRAF–COL4A4*	5 (7.58%)	–	–
	*BRAF–CSMD1*	–	17 (23.61%)	16 (22.86%)
	*BRAF–GPR112*	6 (9.09%)	–	6 (8.57%)
	*BRAF–LCE3C*	–	25 (34.72%)	–
	*BRAF–NBEA*	5 (7.58%)	5 (6.94%)	5 (7.14%)
	*BRAF–NLRP3*	5 (7.58%)	–	–
	*BRAF–PLG*	–	–	5 (7.14%)
	*BRAF–TTN*	9 (13.64%)	–	–
	*BRAF–ZNF234*	5 (7.58%)	–	–
Frequency of *BRAF*-related MM-positive cell lines among total *BRAF-*mutated cell lines		28 (42.42%)	37 (51.39%)	28 (40.00%)
Other gene pairs	*ABCA12–PDZD2*	––	–	5 (0.57%)
	*C7–USH2A*	–	–	5 (0.57%)
	*LRRTM3–SCN1A*	–	5 (0.54%)	–
Frequency of MM-positive cell lines among total drug-tested cell lines		28 (3.25%)	42 (4.50%)	35 (3.97%)

*Number of cell lines with IC_50_ data for each drug available.

**Figure 2:**
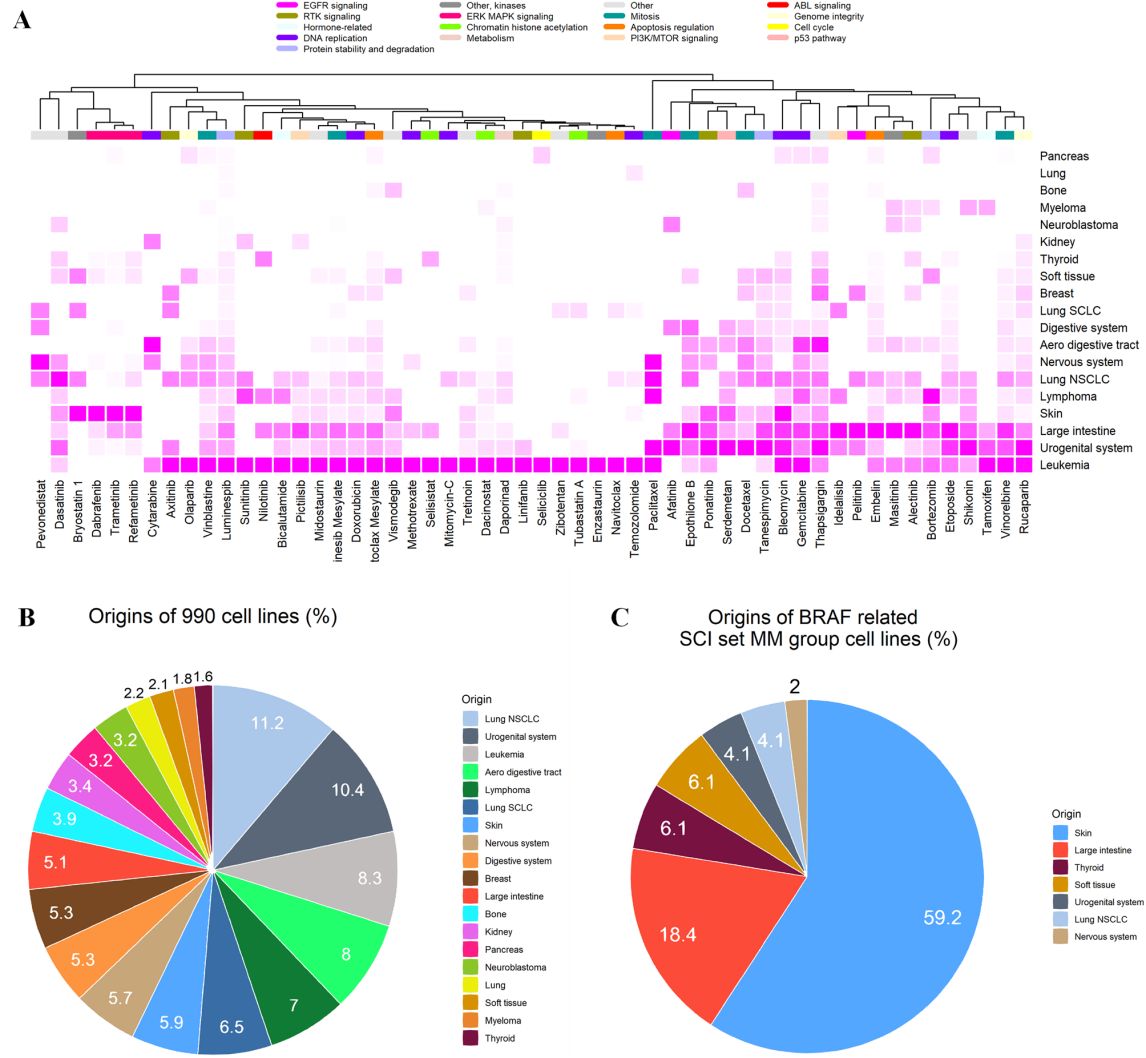
SCS interaction set drug/cell line origin clustering and their tissue origins. (**A**) Heat map of drug/origin tissue hierarchical clustering (rows: drugs, n = 54; columns: origins, n = 19). The complete linkage method and Euclidean distance were used for hierarchical clustering. (**B**,**C**) Pie charts of cell line origins for all 990 cell lines and 49 cell lines for BRAF/MAPK pathway-targeting drug and *BRAF-*related SCS interaction sets in the MM group. Chi-squared tests were applied for the cell line origin count table (rows, n = 19; columns, n = 2). *SCLC* small-cell lung carcinoma, *NSCLC* non-small-cell lung carcinoma.

We further performed clustering analysis of the drugs according to the tissue origin of cell lines to explore the potential of tissue-specific enrichment of SCS interaction sets. The GDSC database classifies the tissue of origin in two ways with different levels of resolution. Using tissue descriptor 1, there were 19 tissue origins of the cell lines according to the anatomical location of cancer occurrence, and 55 origins according to the specific diagnosis or pathological classification of cancer. Using the latter pathological classification, we obtained a stable cluster of three BRAF/MAPK inhibitors that were mostly enriched in melanoma and colorectal cancer cell lines (Fig. 2A). There was a significant difference between the total GDSC cell lines and the BRAF/MAPK inhibitor-related SCS interaction set enriched cell lines (*P* < 0.05). Overall, these results showed that cell lines with SCS interaction sets for BRAF/MAPK inhibitors were significantly enriched with tissues originating from the skin (59.2%, all melanoma) and large intestine (18.4%, all colorectal cancer). The pie charts in Fig. 2B,C visually compare the distribution of the cell lines enriched in SCS interaction sets for BRAF/MAPK inhibitor clusters according to the tissue of origin for the total 990 cell lines and 49 MM group cell lines, respectively. Of the total, 55 cell lines were classified as melanoma (5.56%), and 50 were classified as colorectal cancer (5.05%).

Detailed tissue origin and mutation profile information for the 49 *BRAF*-related MM group cell lines are provided in Supplementary Table S4, and a heat map of BRAF/MAPK pathway-targeting drugs related to the 49 *BRAF-*related MM group cell lines is provided in Supplementary Figure S2.

### Combination effects of partner genes with *BRAF*-related SCS interaction sets for BRAF/MAPK inhibitors

**Figure 3:**
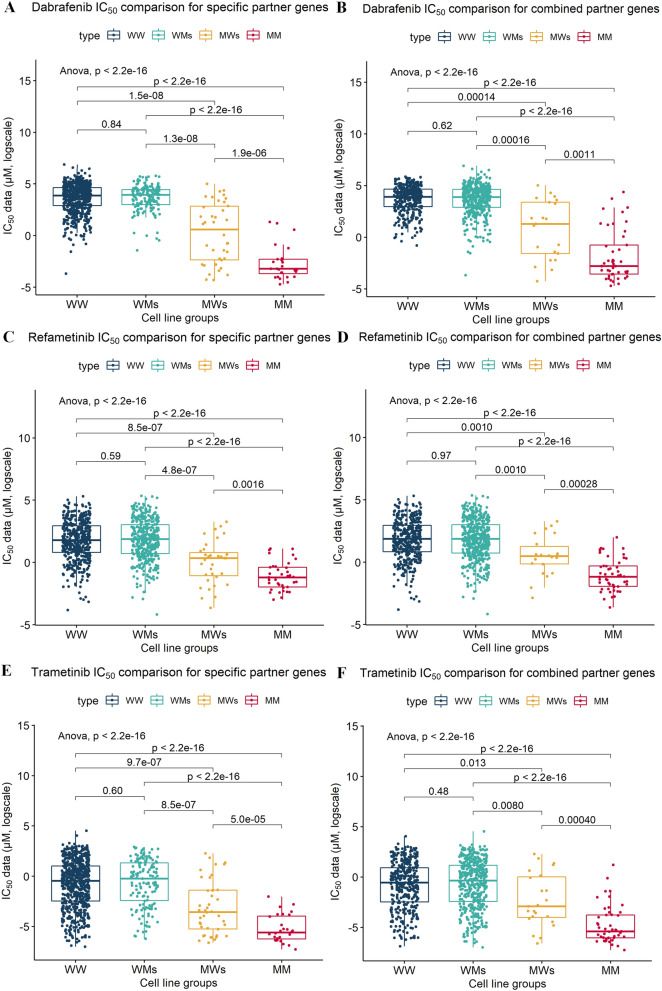
Comparison of drug sensitivity among cell line groups according to the mutational profiles of *BRAF* and *BRAF*-related synergic gene partners for BRAF/MAPK pathway-targeting drugs. IC_50_ values were compared between groups divided according to their mutational status (WW, WM, MW, and MM) and partner gene combination [drug-specific partner genes combined for each drug (**A**,**C**,**E**) or multidrug combined partners for all three drugs (**B**,**D**,**F**)]. Comparisons among WW, WM, MW, and MM groups were performed using Student’s *t* tests. W indicates wild-type genes, and M indicates mutated genes. The first letter indicates the mutational status of BRAF, and the second letter indicates the mutational status of the partner gene(s). For example, in WM, *BRAF* is wild-type, and the partner gene is mutated. *MMC* mutated *BRAF* and mutated combined partner genes, *MWC* mutated *BRAF* and wild-type combined partner genes, *WMC* wild-type *BRAF* and mutated combined partner genes, *WWC* wild-type *BRAF* and wild-type combined partner genes, *s* drug-specific partner genes.

To identify any underlying intergenic component among the partner genes in the *BRAF*-related SCS interaction sets, we combined the partner genes within each drug and across all three BRAF/MAPK inhibitors. The effects of drug-specific combined partner genes were evaluated when a cell line had a damaging mutation in any of the drug-specific partner genes (e.g., *CSMD1*, *LCE3C*, or *NBEA* for refametinib). Such cell lines were classified as the mutated type with regard to the partner genes and were denoted as the drug-specific combined M type. At the same time, all drug combined partner genes included 11 *BRAF*-related SCS interaction partner genes for the three drugs. When a cell line harboured a mutation in any of these genes, it was denoted as multidrug combined M type. Comparisons of the results and distributions of IC_50_ values for the four groups for each drug are shown in Fig. 3A,C,E. Figure 3B,D,F show the combined effects of partners of *BRAF* for the three drugs. In both the drug-specific and three-drug combined comparisons, the combined effects of partners were confirmed as being significant (the maximum *P* values from Student’s *t* tests in the drug-specific combined MW group versus the drug-specific combined MM group was 0.0016, and that in the multidrug combined MW group versus the multidrug combined MM group was 0.0011). All three drugs showed significantly lower IC_50_ values in the mutated cell lines for drug-specific combined partners and across the three drugs for combined partners compared with those in the MM group. Furthermore, the MM group showed the lowest IC_50_ values among the four groups in both combinations for the three drugs. Furthermore, the multidrug combined MM and drug-specific combined MM groups showed the lowest IC_50_ values among the four groups for both combinations for the three drugs.

In addition, we evaluated whether there were additive effects among partner genes for each of the three drugs. To evaluate whether cell lines with large numbers of mutations in partners genes were more sensitive to drug effects, we divided the drug-specific combined MM and multidrug combined MM cell lines according to the number of mutations in their partner genes and compared their IC_50_ values (Supplementary Figure S3). The numbers of mutations in partner genes ranged from one to seven. Because there were very few cell lines with mutations in three or more partner genes for each drug, it was difficult to confirm the statistical significance; however, a decrease in IC_50_ was observed as the number of mutations in the partner genes increased. To further confirm this additive effect, we divided the mutated partner gene cell lines into three groups according to the number of mutations they possessed (zero, one, and two or more) to identify statistically significant differences (Supplementary Figure S4). When comparing the IC_50_ values of cell lines separated by the number of mutations in three BRAF/MAPK inhibitors in both drug-specific and multidrug partner genes combinations, the group with one mutation per partner gene (combined MM1) was significantly lower than that in the other three groups for both multidrug (*p* < 0.023) and drug-specific comparisons (*p* < 0.01), except for dabrafenib when evaluating multidrug combined partners (*p* = 0.095). However, a comparison of IC_50_ values for MM1 (mutated *BRAF* and only one mutated partner gene) versus MM2 or MM3 (mutated *BRAF* and two or three more mutated partner genes) revealed no significant differences in drug reactivity, except for dabrafenib (*p* = 0.034) and trametinib (*p* = 0.025) in the multidrug combined partners comparison.

### Comparison of mutation profiles of the SCS interaction sets and all GDSC cell lines

To confirm whether the BRAF/MAPK pathway-targeting drug-related synergistic interaction set was affected by the basic characteristics of the mutational profile or by the tissue origin of the cell lines, we compared mutation frequencies between the multidrug combined MM cell lines in *BRAF-*related sets and all 990 GDSC cell lines. As shown in Table 2, loss-of-function (LoF) and missense mutations were significantly more frequent (*P* < 0.001) for partner genes with *BRAF* in SCS interaction sets in the multidrug combined MM cell lines, and the copy number variation (CNV) deletion frequency tended to be higher, but the difference was not statistically significant (*P* < 0.1). However, there were no significant differences for all cell lines when considering the three mutation profiles for all genes (*P* > 0.1).

**Table 2:** Statistical comparison of mutation profiles of *BRAF-*related multidrug combined MM cell lines and all 990 cell lines.

	Multidrug combined MM cell lines (n = 49)	All GDSC cell lines (n = 990)	*P* value*
	Mean ± SD	Mean ± SD	
**Partner genes related to BRAF in SCS interaction sets (n = 12)**			
LoF	0.57 ± 0.74	0.13 ± 0.45	< 0.001
Missense	2.02 ± 1.13	0.39 ± 0.89	< 0.001
CNV deletion	0.51 ± 0.51	0.37 ± 0.48	0.064
**All genes (n = 19,100)**			
LoF	29.16 ± 28.65	23.96 ± 57.90	0.251
Missense	125.51 ± 134.66	97.28 ± 183.85	0.166
CNV deletion	20.12 ± 9.03	21.69 ± 13.66	0.254

*Student’s *t* test.

### Survival analysis with pan-cancer cohort data

**Figure 4:**
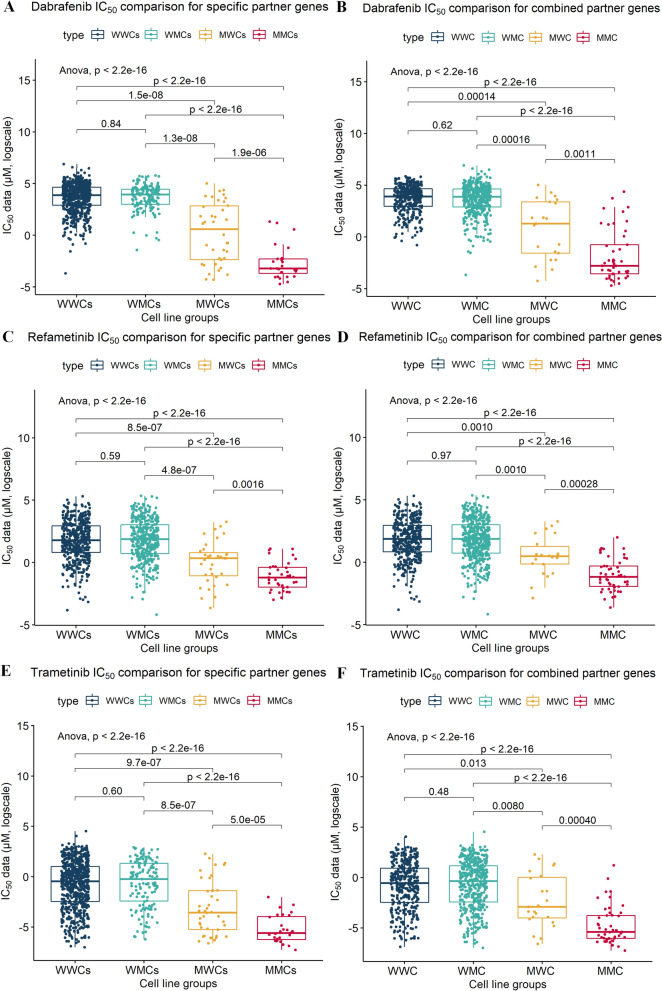
Survival analysis of BRAF inhibitor-treated patients. The 65 BRAF inhibitor-treated patients were separated into four groups according to their mutation profiles: WWC, WMC, MWC, and MMC; W indicates the wild-type gene, M indicates the mutated gene, and C indicates the multidrug combined partner genes. The first capital character denotes the *BRAF* gene, and the latter two capital letters denote the mutational statuses of the multidrug combined partner genes. Log-rank tests were applied to determine the significance of differences in survival.

Among the 65 patients included in the pan-cancer analysis who were treated with BRAF inhibitors, MEK inhibitors, or a RAF inhibitor with available somatic mutation profiles from The Cancer Genome Atlas (TCGA), 30 were in the WW group, 22 were in the WM group, two were in the MW group, and 11 were in the MM group when we combined all partner genes derived from the previous GDSC data analysis (Fig. 4). Survival analysis showed that the patients in the MM group had longer median overall survival (OS) compared with those in the WW group (64.10 versus 27.13 months; hazard ratio [HR] = 0.44, *P* = 0.101) and MW group (64.10 versus 19.15 months; HR = 0.096, *P* = 0.007). The patient count table for the use of BRAF/MAPK inhibitors and gene mutations is shown in Supplementary Table S4. Univariate Cox regression for each factor indicated no significant effects of age, sex, or carcinoma type on survival, whereas mutation type had an effect. Multivariate Cox regression analysis confirmed that only mutation type was a significant independent predictor of survival (*P* = 0.0093).

### Melanoma-specific analysis in GDSC and TCGA data

Both *BRAF*-associated synergistic partners for BRAF inhibitors in GDSC and TCGA data were enriched in melanoma. Therefore, we re-analysed melanoma separately. Among the nine genes identified as BRAF partners shown in Fig. 1, the genes that we were able to confirm statistical significance for in relation to dabrafenib were *C10orf112*, *GRP112*, *NBEA*, *TTN*, and *ZNF234*. For refametinib, only *NBEA* was confirmed to be significant, and for trametinib, *NEBA* and *GPR112* were significant (Supplementary Table S5). In drug-specific/multidrug analyses (Supplementary Figure S5), excluding drug-specific analysis of refametinib (*p* = 0.086), the MM group showed the best drug reactivity (*p* < 0.05).

### Comparison of mutational profiles in resistance genes

Previous studies on treatment responsiveness to BRAF/MAPK inhibitors have primarily focused on the presence of mutational profiles associated with drug resistance. To confirm that the synergistic sensitivity of our identified genetic profile was not a secondary consequence of mutations in genes previously associated with BRAF inhibitor resistance, we compared only mutations in well-known resistance genes, including *BRAF* amplification and mutations in genes of the MAPK family (including *MEK1* and *MEK2*), *PTEN*, *RAC1*, *CDK4*, *AKT1*, *NF1*, and *NRAS*^10–14^. In the GDSC database, 194 cell lines from 936 patients (20.6%) had at least one resistance alteration. Fifteen of the 62 patients analysed (23.1%) in TCGA database had at least one of the resistance-associated alterations. When we divided cell lines and patients according to the mutational status of *BRAF* and *BRAF*-related multi-drug combined partner genes, there were no significant differences in the proportion of patients harbouring likely resistance alterations among the four groups (WW, WM, MW, and MM) in the GDSC and TCGA data (Supplementary Tables S6, S7, and S8). In addition, chi-squared tests showed no significant differences in the presence or absence of well-known BRAF/MAPK inhibitor resistance gene mutations between the multidrug combined MM and multidrug combined MW groups for both the TCGA and GDSC cell lines (*P* = 1).

### Comparison of the expression profiles of the candidate SCS interaction partners in the GDSC database

**Figure 5:**
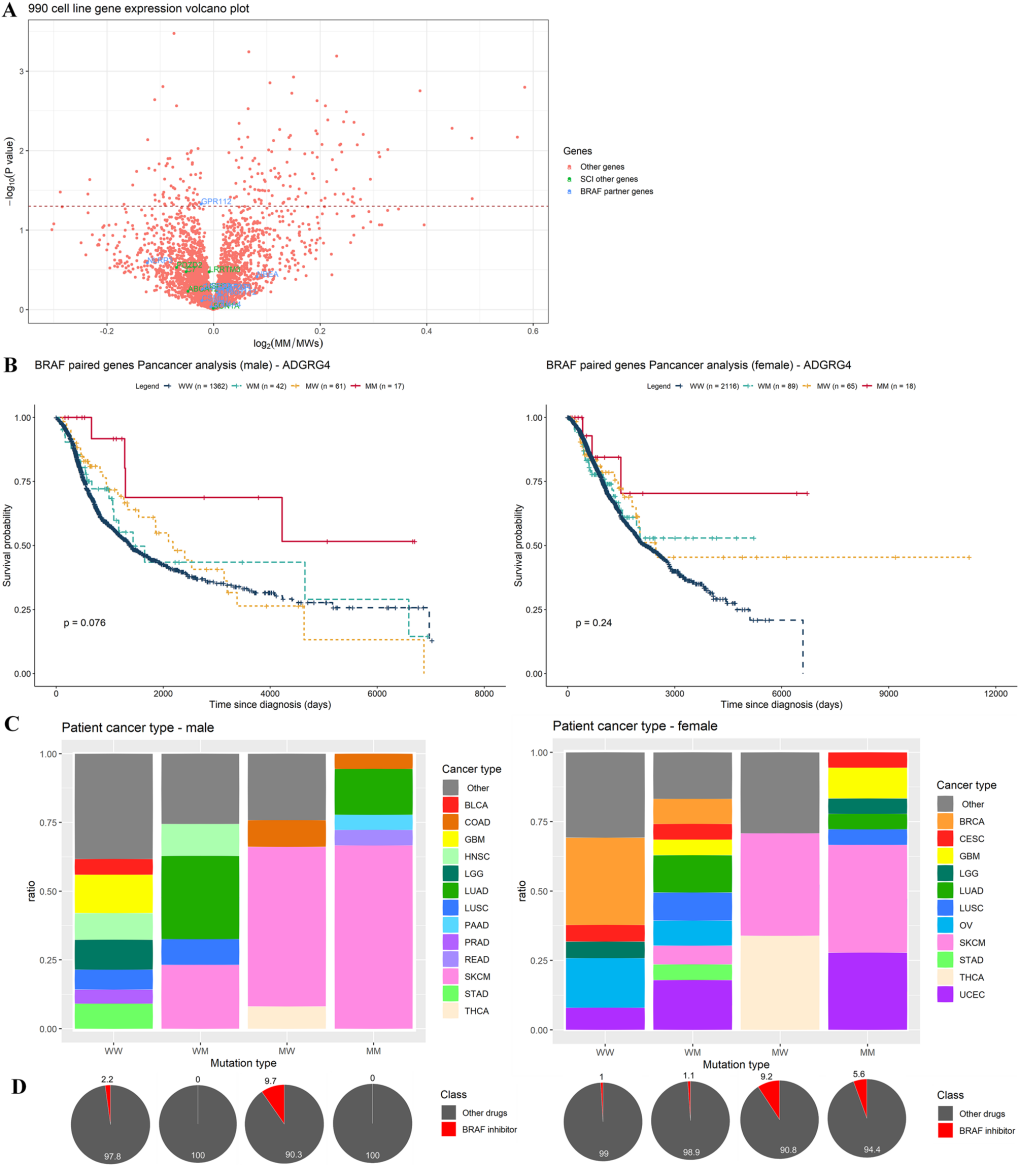
GDSC and TCGA analysis of *BRAF* and *GPR112* (*ADGRG4*) coexistence. (**A**) Volcano plot of gene expression profiles between the MM and MW groups for the 990 GDSC cell lines and 3804 filtered genes. (**B**) Sex-specific survival analysis of pan-cancer patients in TCGA database, including 1482 males (left) and 2288 females (right). Patients were separated according to the mutation profiles of *BRAF* and *GPR112* (*ADGRG4*). (**C**) Sex-specific stacked bar charts according to cancer type (left, males; right, females). Cancer types with a proportion less than 5% were included in the “other” group, including 27 and 25 cancer types for males and females, respectively. (**D**) Sex-specific pie chart for the use of BRAF inhibitors. Left, males; right, females.

We next sought to identify partner genes of the *BRAF*-related SCS interaction set with the greatest contributions. Therefore, we compared the expression profiles of all candidate partner genes in GDSC cell lines. Among cell lines with *BRAF* V600E mutations, those simultaneously carrying mutations in one or more of the 11 partner genes were grouped into the multidrug combined MM group and those containing only *BRAF* mutations were grouped into the multidrug combined MW group. Among these, only *GPR112* (*ADGR4*) showed significantly lower expression in MM cell lines than in MW cell lines (*P* = 0.047; Fig. 5A).

To evaluate whether there was a hidden interaction effect between *BRAF* and *GPR112*, we included patients who had mutational profiles of both *BRAF* and *GPR112* from TCGA database. The 3770 patients were then divided into four groups according to the coexistence of *BRAF* and *GPR112*, for survival analysis. Patients in the MM group had longer median OS compared with those in the WW group (35.70 versus 24.67 months; HR = 0.37, *P* = 0.009) and MW group, that is patients with mutated *BRAF* but wild-type *GPR112* (35.70 versus 31.85 months; HR = 0.45, *P* = 0.050). In both the univariate and multivariate Cox regression analyses, age, sex, and mutation profile all affected survival (*P* < 0.05), whereas carcinoma type did not (*P* > 0.05). Because of the impact of sex on patient survival, we conducted a stratified survival analysis according to sex. For both males (n = 1482) and females (n = 2288), patients in the MM group also had longer median OS compared with those in the WW group (males: 40.97 versus 21.40 months, HR = 0.33, *P* = 0.026; females: 27.17 versus 26.97 months, HR = 0.42, *P* = 0.131) and MW group (males: 40.97 versus 33.4 months, HR = 0.40, *P* = 0.084; females: 27.17 versus 31.33 months, HR = 0.54, *P* = 0.327). Both univariate and multivariate Cox regression analysis showed that age significantly affected survival, whereas carcinoma type and mutation profile did not for both males and females (Fig. 5B). Therefore, the sex-specific survival analysis did not differ from the all-patient analysis. However, when we focused on melanoma type only, the survival differences according to *ADGRG4* gene status and sex disappeared, as shown in Supplementary Figure S6. Cancer type and BRAF/MAPK inhibitor use status exhibited different distributions between males and females. *GPR112* was a variable that was related to significant differences in survival when survival analysis was performed without sex classification; however, the difference disappeared when survival analysis was performed after adjustment for sex. This may be because carcinoma type varies significantly according to sex (Fig. 5C) and because age, mortality, and molecular genetic profiles (Fig. 5D) also vary depending on carcinoma type. Sex-specific distributions for patients with different cancer types and BRAF*/*MAPK inhibitors are shown in Fig. 5C,D, respectively.

## Discussion

In this study, we identified gene sets demonstrating synergistic effects with the gain-of-function mutation V600E of the *BRAF* gene in response to BRAF/MAPK inhibitors. This synergistic effect was confirmed in both a cancer cell line database and TCGA cohort. The majority of cases involved melanoma (59.2%) and colon cancer (18.4%) in the GDSC database and only patients with melanoma from TCGA. This hypothesis-free approach identified several meaningful candidate synergistic drug response interaction sets in the GDSC database, including 11 genes showing synergistic relationships with a *BRAF* mutation for three common BRAF/MAPK inhibitors (dabrafenib, refametinib, and trametinib): *C10orf112* (*MALRD1*), *COL2A1*, *COL4A4*, *CSMD1*, *GPR112* (*ADGRG4*), *LCE3C*, *NBEA*, *NLRP3*, *PLG*, *TTN*, and *ZNF234*. Moreover, survival analysis of patients taking BRAF/MAPK inhibitors with available data from TCGA showed a significant survival advantage for patients with mutations in both *BRAF* and partner genes. Mutations known to be associated with resistance to BRAF/MAPK inhibitors, including *BRAF* amplification and mutations in genes of the MAPK family (*PTEN*, *RAC1*, *CDK4*, *AKT1*, *NF1*, and *NRAS*)^10–14^, were not significantly enriched in cell lines with mutations in both *BRAF* and its partner genes or in patients from TCGA data. Overall, our results suggested that certain molecular profiles represent a synergistic response with *BRAF* mutation to BRAF/MAPK inhibitors, highlighting these mutation signatures as worthy candidates for further study.

Our study validated the existing conditional synthetic lethality concept, i.e., variations of two genes increases drug responsiveness, in published drug treatment cell line databases and in large-scale cancer cohorts. Traditional SL studies have been conducted mainly from two perspectives: predicted interactions and unexpected connections^6^. Research using predicted interactions (i.e., the concept that key genes belonging to two parallel pathways involved in survival-related functions contribute to synthetic lethal interactions) is already being actively studied. There is even a database that comprehensively collects and provides relevant information^15^. However, the mechanisms involved in the survival and growth of cancerous tissues are quite different from those of normal tissues. Changes in the tumour microenvironment, which occur constantly with tumour growth, in addition to chemotherapy and radiation therapy, force cancer cells to evolve in a wide variety of ways^16^. Thus, given our current lack of knowledge about the functions and roles of various genes, the discovery of drug targets in SL, which relies entirely on known mechanisms, is also limited. The *BRAF*-associated target therapy sensitivity genes derived from our study have a wide variety of known roles and functions; however, their associations with the BRAF/MAPK pathway are not clear. Despite this, the effects of target therapy inhibitor sensitivity exhibited by these genes have been clearly identified, suggesting they may be useful in the fight against cancer.

*BRAF* encodes BRAF protein, a member of the Raf kinase family of growth signal transduction protein kinases^17^. BRAF plays a role in regulating the MAPK/ERK signalling pathway, which is related to cell division, secretion, and differentiation. *BRAF* mutations occur in more than 50% of cutaneous melanomas, with *BRAF* V600E being the most common; this mutation results in constitutive monomeric activation of BRAF kinase activity^18^. Targeted therapy with BRAF/MAPK inhibitors for mutated BRAF proteins significantly improves survival. Among the BRAF/MAPK inhibitor-treated cell lines in the GDSC database, approximately 7% had *BRAF* mutations, and 40–50% of these had variations in all of the partner genes in the *BRAF*-related synergistic interaction sets. Assuming that all 11 candidate SCS interaction partner-genes showing synergistic relationships to BRAF/MAPK inhibitors in *BRAF*-mutated cells were true positive signals, then more than 40% of all *BRAF*-mutated cells had at least one interacting partner mediating the drug response. This finding was relatively consistent with previous studies showing that mutations in genes involved in putative BRAF resistance mechanisms, such as *NRAS*, *KRAS*, *BRAF* splicing variants, *BRAF* amplification, and *MEK*, could explain 39–42% of the resistance to BRAF inhibitor therapy^10,14,19–21^. Furthermore, we did not find mutations in genes previously associated with BRAF/MAPK resistance in cell lines with one or more mutations in the *BRAF* and SCS interaction partners-genes. Analysis of the GDSC database identified *C10orf112* (*MALRD1*), *COL2A1*, *CSMD1*, *GPR112* (*ADGRG4*), and *NBEA* as SCS interaction genes common to at least two BRAF/MEK inhibitors. Because *C10orf112*, *CSMD1*, and *NBEA* are mutated in most cancers at frequencies of 90–100%, they are suggested to be passenger genes^22^. However, these genes showed more sensitive responses to BRAF/MAPK inhibitors in cell lines with more than one mutation; this suggests that not only the function of individual genes but also unknown underlying combinatorial effects or interactions which exist between these genes may influence drug sensitivity.

Our study suggested that *GPR112* may be a very promising drug target. *COL2A1* and *GPR112* have high mutation rates in melanoma, but their roles differ in other types of carcinomas. In particular, *GPR112*, which encodes a G-protein coupled receptor (GPCR), is considered a potential cancer treatment target^23^. GPCRs can activate epidermal growth factor receptor (EGFR) either directly or through cleavage of membrane-bound EGFR-ligand precursors, independent of the ligand-binding EGFR activation process. EGFR and its respective ligands are overexpressed in various tumours, and this overexpression is correlated with poor prognosis in certain cancers^24^. Previous studies have indicated that the effectiveness of anti-EGFR antibody treatment in patients with *BRAF*-mutated colorectal cancer is lower than that in patients with wild-type *BRAF*. However, this effect was not detected in the subgroup with MAPK-activating mutations in *BRAF* or *RAS* mutations because the anti-EGFR antibody inhibits the MAPK pathway activated by EGFR^25^. This is a possible explanation for the synergistic effects of these genes on BRAF inhibitors as a type of GPCR that can be activated. Additional studies are needed to further support this relationship. For example, single-cell studies, which have recently been used in various disease models, could help us to evaluate whether the gene–gene interactions found in our study are actually caused by the increased burden of pathway interdependence in a single cell or by microenvironmental phenomena, which has been proposed as a supporting hypothesis for the SL concept. The main limitation of our study was that we could not prove causality; that is, further studies are needed to determine whether the synergistic gene sets including *BRAF* truly affect drug reactivity with BRAF/MAPK inhibitors in a manner depending on partner gene mutations using *BRAF-*mutated cell lines. Further experiments should also confirm whether all partner genes identified in our gene set showed true positive associations or are instead neutral passenger genes. Nevertheless, our study is the first to propose a gene set showing synergistic sensitivity to BRAF/MAPK inhibitors in both *BRAF* mutant cell lines and patients using a hypothesis-free approach. With the successful implementation of various molecular biological profiles of cancer, a variety of therapeutic approaches have been developed and tested, including customised cancer therapies and immunotherapies, with some good results. However, treatments using cancer-specific genetic characteristics have not yet been developed and applied in clinical trials. Therefore, in addition to developing new therapies, identifying new targets to which existing therapies can be applied and identifying subpopulations who may benefit from treatment are important tasks in improving cancer treatment. Since we demonstrated that the mutational status of gene pairs can successfully predict drug sensitivity in cell lines as well as patient survival, these gene pairs are worthy candidates for selection of an effective treatment target.

## Methods

### Datasets

**Figure 6:**
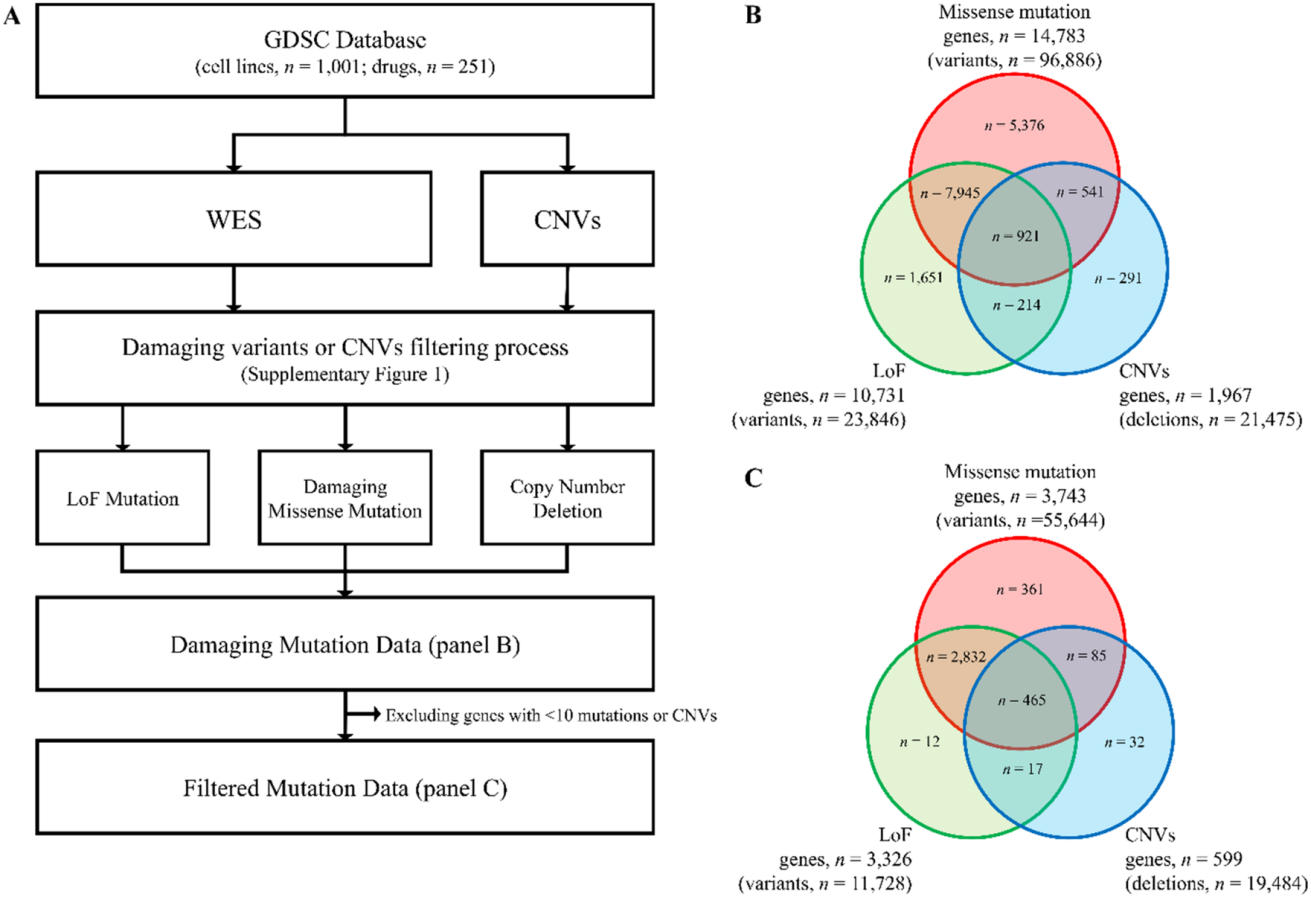
Schematic diagrams of the data selection process to identify damaging mutations. (**A**) Flowchart to identify and filter damaging mutations from the molecular profiles annotated by the GDSC curation team divided into two types: coding variants and copy number alterations. To identify damaging variants, loss-of-function mutations (stop-loss and nonsense variants) and damaging missense mutations (variants predicted to cause damage by SIFT and PolyPhen scores) were extracted from the coding variant annotation data. Copy number deletions were extracted from the copy number alteration data. The filtering process of damaging variants/deletions is shown in Supplementary Figure S5. (**B**) Venn diagram of the damaging variants/deletions before filtering. (**C**) Final gene set used in the analysis after filtering out genes with less than 10 damaging mutations (n = 3804). *GDSC* Genomics of Drug Sensitivity in Cancer, *CNV* copy number variation, *LoF* loss-of-function mutation.

We first screened for candidate gene pairs showing synergistic drug-response using the GDSC database, consisting of genetic profiles for 1001 cancer cell lines and drug sensitivity profiles for 251 chemicals^26^ (https://www.cancerrxgene.org/downloads, accessed 21 March 2019). The GDSC project released molecular profile data, including drug sensitivity (IC_50_ values) and cell line molecular profiling data. CNVs, whole-exome sequencing (WES)-based variants, DNA methylation, and gene expression data are available in this database as part of the cell line molecular profiles. Among these data, we collected drug sensitivity data from pretreated cell lines, CNV data, and WES variant data. Among the WES variants, we extracted only the mutations known or predicted to be damaging based on the functional annotation classification of mutation information provided by the GDSC database. For the CNV data, we extracted only homozygous deletions from the annotation classification provided by the GDSC database, as shown in Fig. 6.

Based on these criteria, data were available for 990 cell lines, and a drug sensitivity response (reported as the IC_50_ value) was available for 251 drugs. The IC_50_ value of the drugs was used as the marker to determine whether a given cell line showed greater sensitivity to drugs when two damaging genes were mutated compared with lines with only one or none of the genes harbouring a damaging mutation. Since the IC_50_ data were not available for all drugs in all 990 cell lines, the arithmetic means of raw IC_50_ (μM) values (natural log scale) were used for analysis of 11,664 replicated drug-cell line pairs. Most drugs were tested once; however, some were tested at two different laboratories. In those cases, we used the arithmetic mean of both IC_50_ values. No cases were replicated three or more times. Drug information data were obtained from the GDSC screened compounds database^26^.

For survival analysis, we used TCGA data obtained from the Bioconductor^27^ (version 3.9) software package TCGABiolinks^28–30^ (version 2.12.5), generated by TCGA Research Network (https://www.cancer.gov/tcga). Somatic mutation data and clinical data, including chemotherapy information, downloaded from 33 TCGA cancer projects for pan-cancer analyses were used in the current analysis (downloaded on 20 August 2019).

### Mutational profile analysis in cancer cell lines

To find SCS interaction gene pairs for cancer drugs, we only considered mutations that were significantly damaging, defined as a gene with one or more LoF mutations and/or missense mutations filtered according to SIFT^31^ and PolyPhen-2^32^ scores and/or copy number deletions. Mutation data were extracted from preprocessed WES data in the GDSC database^26,33^. Schematic diagrams of the datasets and filtering procedure used for this analysis are shown in Fig. 6, and the detailed process is outlined in Supplementary Figure S5. Stop-loss and nonsense classifications from WES data were considered damaging LoF mutations (n = 23,846). To define damaging missense mutations, SIFT and PolyPhen scores of missense mutations were mapped using Ensembl Variant Effect Predictor^34^ (https://grch37.ensembl.org/Multi/Tools/VEP, accessed on 7 May 2019). GRCh37 was used as a reference genome for the WES data. Only transcripts representing Ensembl ID (ENST)-tagged data were included in the analysis, and both deleterious (SIFT score < 0.05) and probably damaging (PolyPhen score > 0.908) missense mutations in the WES data were considered damaging missense mutations (n = 96,886). CNV data were obtained from the gene-level copy number data of GDSC (accessed on 21 March 2019). A homozygous deletion was considered to be a copy number deletion (n = 21,475). A gene/cell line mutation count table was constructed for the LoF mutations, damaging missense mutations, copy number deletions, and assembled data of these three categories, including a total of 19,100 genes in 990 cell lines (Supplementary Table S9). Genes with a damaging mutation in less than 10 cell lines were excluded from the analysis. After filtering, 3804 genes (7,233,306 gene–gene pairs) remained.

### Drug sensitivity analysis to identify SCS interaction sets

SCS interaction sets were identified by determining the relationships of the IC_50_ values of each drug and the mutation profiles of the cell lines. Pairwise comparisons were performed for all gene pairs for each of the 251 drugs to identify candidate SCS interaction sets without an underlying hypothesis of any specific drug/gene–gene interactions. Using the 3804 genes included from the previous filtering steps (Fig. 6), the cell lines were divided into four groups according to the mutational types of the pair of genes: WW, MW, WM, and MM, where W indicates the wild-type and M indicates a damaging mutation in the gene. Therefore, the two letters represent two genes that are both wild-type, both mutated, or one mutated and the other wild-type. If all four groups for a specific gene pair (*A* and *B*) matched to a given drug, D, contained data for more than five cell lines, these genes were retained for the analysis, and the means of natural log-transformed IC_50_ values were compared between the MM group and the other three groups. Because the number of tested cell lines with IC_50_ values for a specific drug varied among the drugs, the four groups of mutational types also varied among the drugs. Therefore, we started the analysis by selecting a drug D and then separated the cell lines with IC_50_ values for that drug into four groups according to the mutation profile. If the MM group had a significantly lower IC_50_ value than the other three groups, this was considered to represent a “synergistic chemotherapeutic effect” to establish a candidate SCS interaction set. Only SCS interaction sets that satisfied the condition in which the maximum IC_50_ value of the MM group was larger than the median IC_50_ value of the WW group were included for further analysis. Once this meaningful cluster of SCS interaction sets was identified, the partner genes of the interaction gene pairs were grouped and then the burden effects of combined gene sets on the synergistic chemotherapeutic response were compared. That is, if gene *B* recurrently appeared in the interaction sets for a specific drug, *D,* and there were multiple genes related to gene *B* among the identified SCS interaction pairs, we combined all of the partner genes of gene *B* and divided the cell lines into the following groups according to their mutation status for *B* and its partners: drug-specific combined WW, drug-specific combined MW, drug-specific combined WM, drug-specific combined MM. For example, drug specific combined WM would indicate a group of cell lines with damaging mutations in any of the partner genes of gene *B* but not in gene *B* itself.

### Clustering analysis of candidate SCS interaction sets

To cluster the interaction sets according to drugs, we first classified the drugs based on their common consensus target protein or pathway according to the GDSC classification. To identify any relationship between the SCS interaction sets and cell lines in the MM group, hierarchical clustering analysis was performed for the drug and the tissue origin of the cell lines. The complete linkage method and Euclidean distance were used for all hierarchical clustering. The final clustering table was constructed with the drug-wise normalisation of the count table and a range of [0, 1]. For drug–gene clustering, a gene (rows)–drug (columns) binary table was established as the cluster table. However, only genes represented two or more times in the SCS interaction sets were included in the table.

### Survival analysis with TCGA patient data

Based on the main results for SCS interaction clustering, the relationships of the interaction sets with patient survival were limited to patients who had been treated with BRAF/MAPK inhibitors (dabrafenib, sorafenib, trametinib, vemurafenib, and selumetinib [AZD6244]) from chemotherapy clinical TCGA data. In total, 80 patients received chemotherapy with one of these five BRAF/MAPK inhibitors. Somatic mutation data for these patients were downloaded from 12 TCGA cancer projects (ACC, COAD, GBM, KICH, KIRC, KIRP, LIHC, LGG, LUSC, SARC, SKCM, UCEC); these data were available for 65 of 80 patients. Missense mutations, nonsense mutations, splice-site mutations, and translation start site mutations were considered damaging mutations. The 65 patients with both chemotherapy clinical data and somatic mutation data were divided into four groups according to the mutation profile of *BRAF* and its combined partners in SCS interaction in the same manner as described above: multidrug combined WW, multidrug combined WM, multidrug combined MW, and multidrug combined MM, in which the first letter indicates the mutational status of *BRAF* and the second letter indicates any gene in the combined partner genes that is mutated or wild-type. For these four groups, the log-rank test was applied to determine differences in survival rates. In addition, univariate and multivariate Cox proportional-hazards models, including age, sex, cancer type (skin cutaneous melanoma or not), and mutation profiles as factors, were evaluated to determine the main factors contributing to survival differences among the groups.

Survival analysis was also performed for patients included in pan-cancer analyses. Patients were separated into four groups according to mutation profiles for the *BRAF* and *GPR112* genes to determine whether there were significant survival differences when both genes were impaired (MM). For this analysis, somatic mutation data were downloaded from 33 TCGA cancer projects (ACC, BLCA, BRCA, CESC, CHOL, COAD, DLBC, ESCA, GBM, HNSC, KICH, KIRC, KIRP, LAML, LGG, LIHC, LUAD, LUSC, MESO, OV, PAAD, PCPG, PRAD, READ, SARC, SKCM, STAD, TGCT, THCA, THYM, UCEC, UCS, and UVM), including 3781 patients. Missense mutations, nonsense mutations, splice-site mutations, and translation start site mutations were considered damaging mutations. These patients were divided into four groups (WW, WM, MW, and MM) according to the mutation profiles of *BRAF* and *GPR112*. Survival was analysed in the same manner as described above.

### Statistical analysis

In the drug sensitivity analysis, Student’s *t* tests were used for comparing the IC_50_ values of the MM groups (including multidrug combined MM/drug-specific combined MM) and other groups with false discovery rate (FDR) correction such that the model *P* value was defined as the *P* value from Student’s *t* tests for between-group MM and non-MM comparisons (non-MM groups were WW, MW, and WM groups). The drug-wise Benjamini–Hochberg procedure was applied on model *P* values for multiple testing correction, and the adjusted model *P* value was defined as the *Q* value. For each drug, Benjamini–Hochberg multiple testing correction was used to estimate the FDR in the comparison between MM and non-MM groups; the maximum IC_50_ value of the MM group was compared with the median IC_50_ value of the WW group. Chi-squared tests were used for comparing cell line origin ratios and differences in the presence or absence of BRAF/MAPK inhibitor gene mutations between the MM and MW groups. Student’s *t* tests and analysis of variance (ANOVA) were used for comparing groups with respect to the burden effect. For survival analysis, the survival function of TCGAanalyze, a survival analysis tool of R package TCGAbiolinks^35^, was used to compare patient survival among the multidrug combined WW, multidrug combined WM, multidrug combined MW, and multidrug combined MM groups. Differences with *P* values of less than 0.05 were considered significant in all analyses. Most of the statistical analyses were performed in R (version 3.5.0)^36^ and Python (version 3.7.2)^37^. Source codes are available upon request.

## Supplementary information

Supplementary Information.

## Data Availability

We used public data, including GSDS and TCGA. GDSC data were accessed through the database webpage (https://www.cancerrxgene.org/downloads/drug_data), and TCGA data were accessed through the Bioconductor package of TCGAbiolinks (https://bioconductor.org/packages/release/bioc/html/TCGAbiolinks.html).
