# Supplementary material for: Identification of synthetic chemosensitivity genes paired with BRAF for BRAF/MAPK inhibitors
Source: Sci Rep. 2020 Nov 17;10:20001. doi: 10.1038/s41598-020-76909-2 (PMC7672081; doi:10.1038/s41598-020-76909-2)
Supplement: Supplementary file 1 — Supplementary Information. [file 41598_2020_76909_MOESM1_ESM.pdf]

Identification of synthetic chemosensitivity genes paired with *BRAF* for *BRAF*/MAPK inhibitors

Kye Hwa Lee[1,†,*], Jinmin Goh[2,3,†], Yi-Jun Kim[1], and Kwangsoo Kim[4,*]

[1]Centre for Precision Medicine, Seoul National University Hospital, Seoul 03080, South Korea

[2]Biomedical Research Institute, Seoul National University Hospital, Seoul 03080, South Korea

[3]Department of Chemical Engineering, Pohang University of Science and Technology (POSTECH), Pohang 37673, South Korea

[4]Division of Clinical Bioinformatics, Seoul National University Hospital, Seoul 03080, South Korea

* Corresponding authors (geffa@snu.ac.kr, kksoo716@gmail.com)

[†] These authors contributed equally to this work.

Supplementary Materials

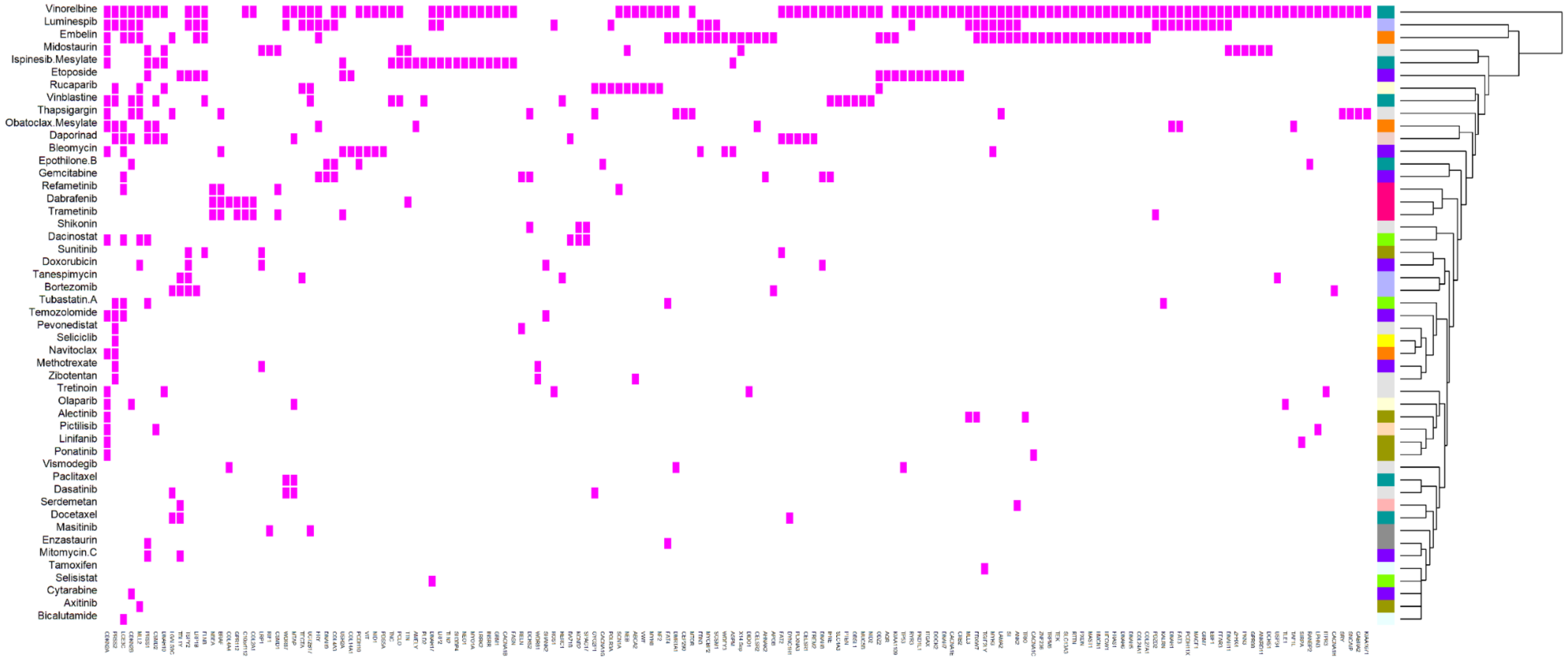

Supplementary Figure S1 | Clustering analysis of the relationship between the drugs and genes of *BRAF*-related SCS interaction sets.
Gene – drug binary table (row: gene, n = 156; column: drug, n = 49) from SCS sets was used as cluster table. Genes appeared two or more times at SCI set were used for clustering. Complete linkage method was processed based on origin of cell line.

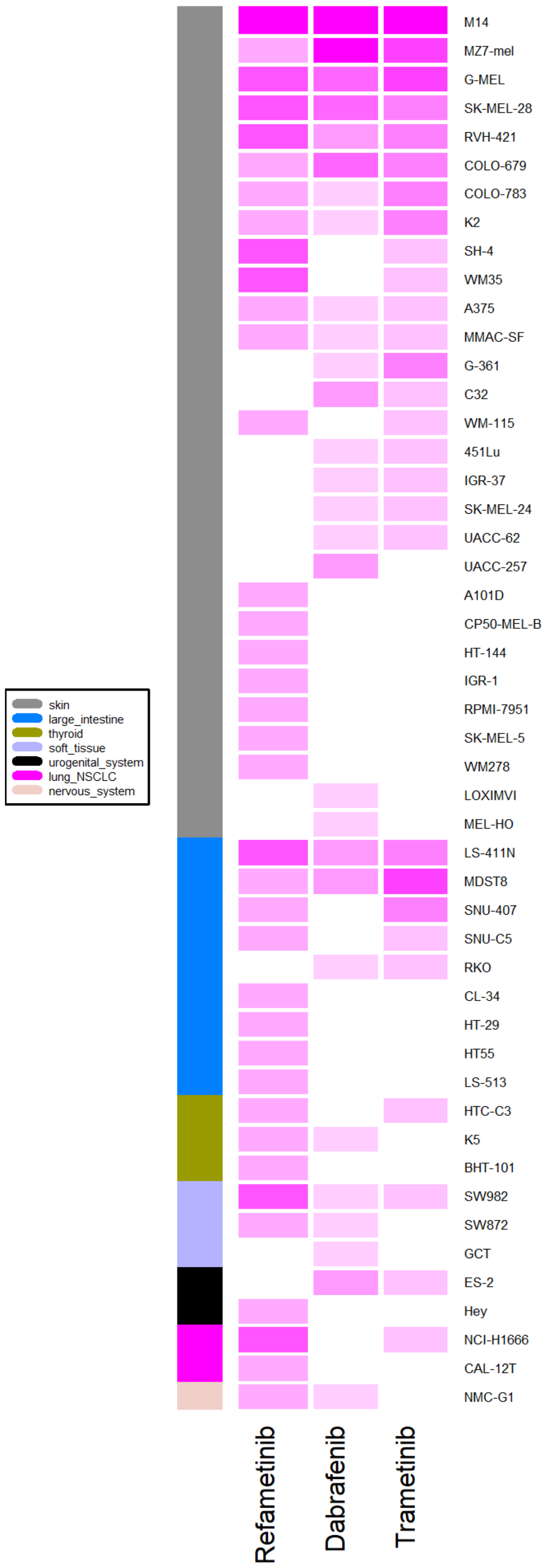

Supplementary Figure S2 | Heat map of *BRAF* related MM group cell lines of ERK/MAPK pathway targeting drugs. Heat map (row: cell lines, n = 49; column: drugs, n = 3) shows the number of cell lines with MM group in SCS sets. We normalized the data by drug-wise. Cell lines are sorted as cell line origin and sum of row.

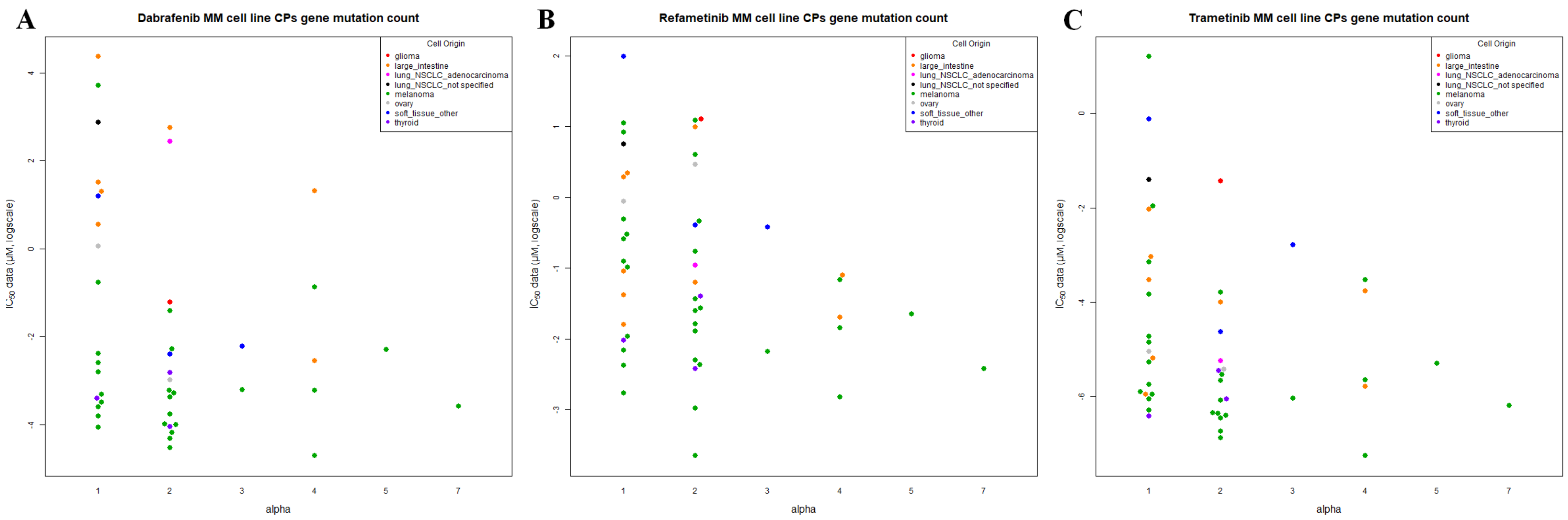

Supplementary Figure S3 | Swarm plot of IC$_{50}$ of *BRAF* related MM group cell lines. A, B, and **C** represents IC$_{50}$ of dabrafenib, refametinib, and trametinib, respectively. Cell lines are separated based on number of mutated αC genes which represent multi-drug combined partner genes. Color mapping and Euclidian distance was used for hierarchical clustering. Only ERK/MAPK signaling pathway targeting drug kept being grouped.

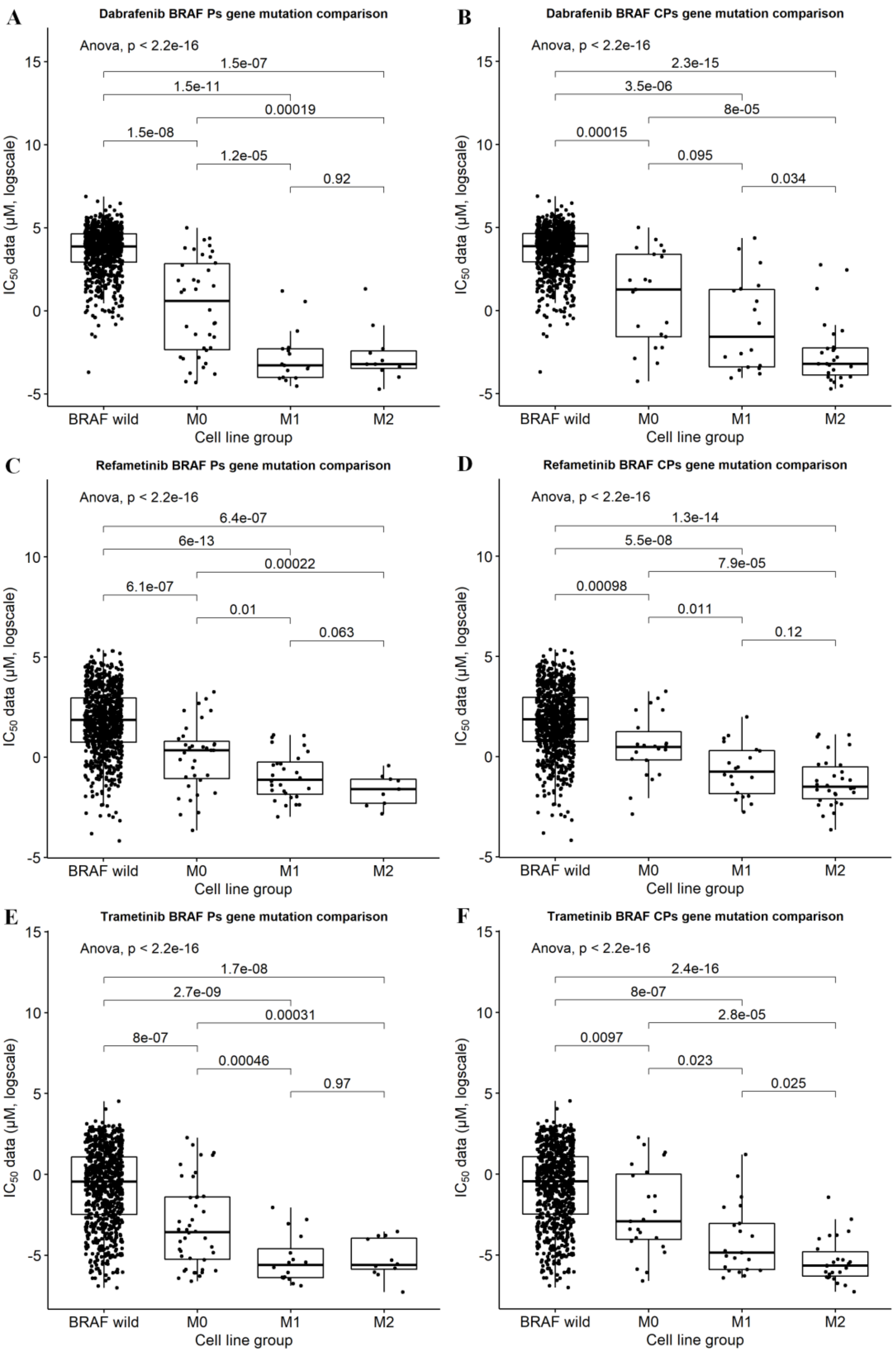

Supplementary Figure S4 | Checking burden effect of *α* genes and *α*C genes with *BRAF* in ERK/MAPK pathway targeting drug. Statistical test was processed for both *α* gene group(drug specific, **A, C, E**) and *α*C gene group(combined, **B, D, F**) base separated cell line groups; *BRAF* wild, M0, M1, M2. All statistical test were Student's t-test. Cell lines of *BRAF* wild group have wild type *BRAF*. Cell lines of M0 group have mutated *BRAF* and wild type *α* or *α*C genes. Cell lines of M1 group has mutated *BRAF* and one mutated *α* or *α*C gene. Cell lines of M2 group has mutated *BRAF* and two or more mutated *α* or *α*C genes.

α gene group: Drug specific group of *α* genes paired with *BRAF*; *α*C gene group: Combined *α* genes for all three ERK/MAPK pathway targeting drugs.

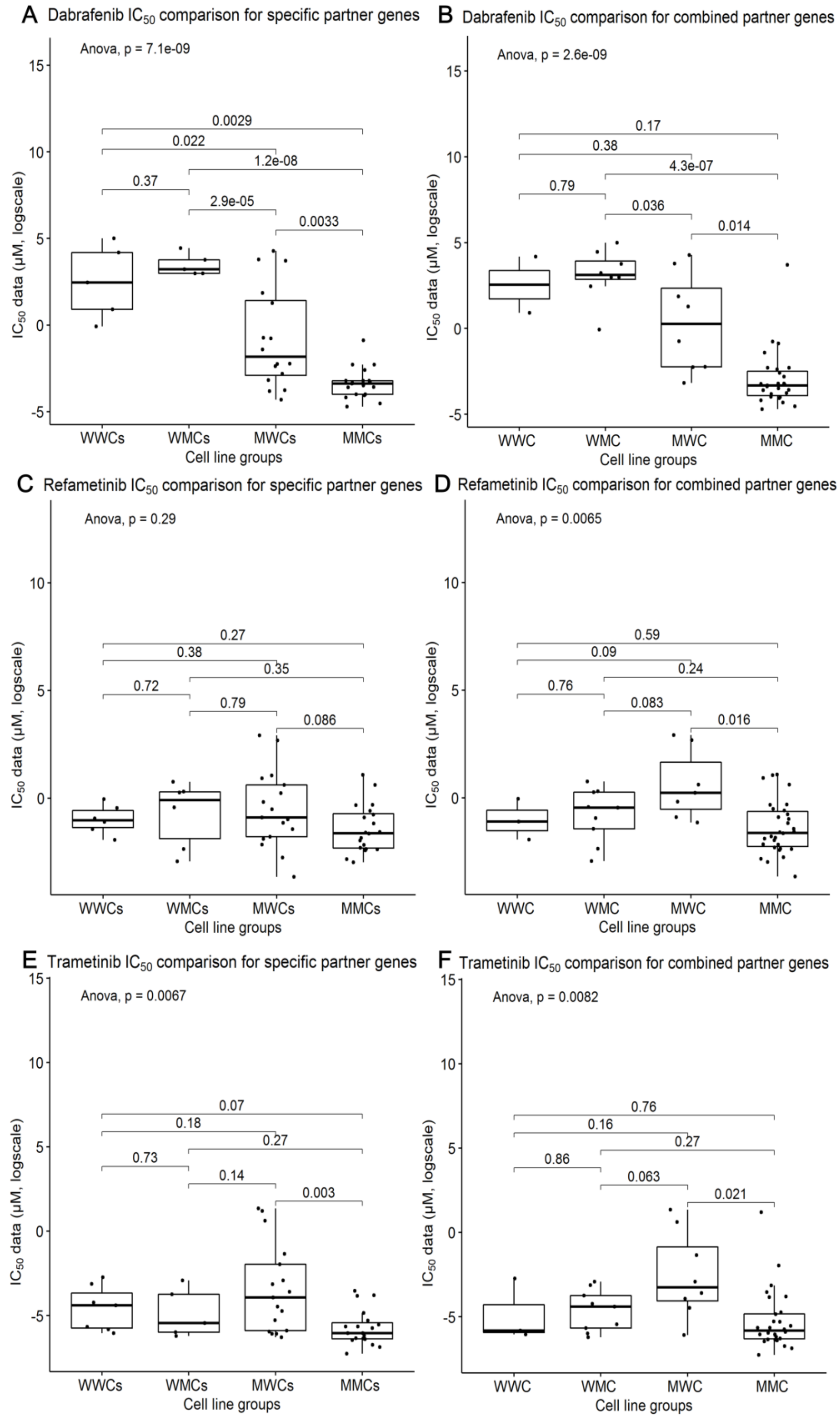

Supplementary Figure S5 | Melanoma-specific analysis for the nine partner genes in BRAF-related SCI sets. Student's t-test was processed for both drug specific gene group (drug specific, A, C, E) and multi-drug combined gene group (combined, B, D, F) base separated cell line groups. The numbers represent p values.

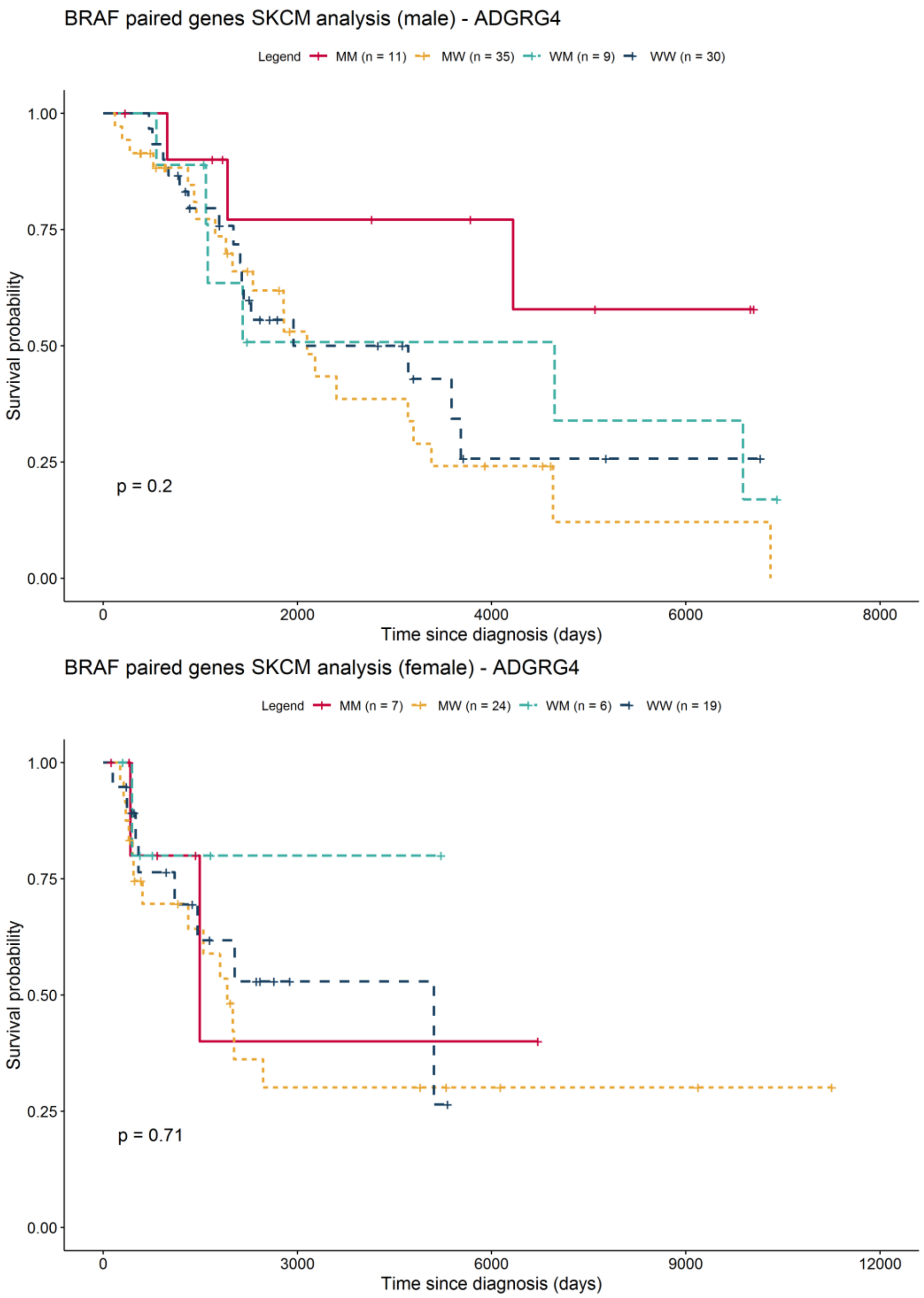

Supplementary Figure S6 | Gender-specific survival analysis of pan-cancer patients in the TCGA
In this analysis, we only included melanoma of 85 males (left) and 56 females (right). Patients were separated according to the mutation profiles of *BRAF* and *GPR112* (*ADGRG4*).

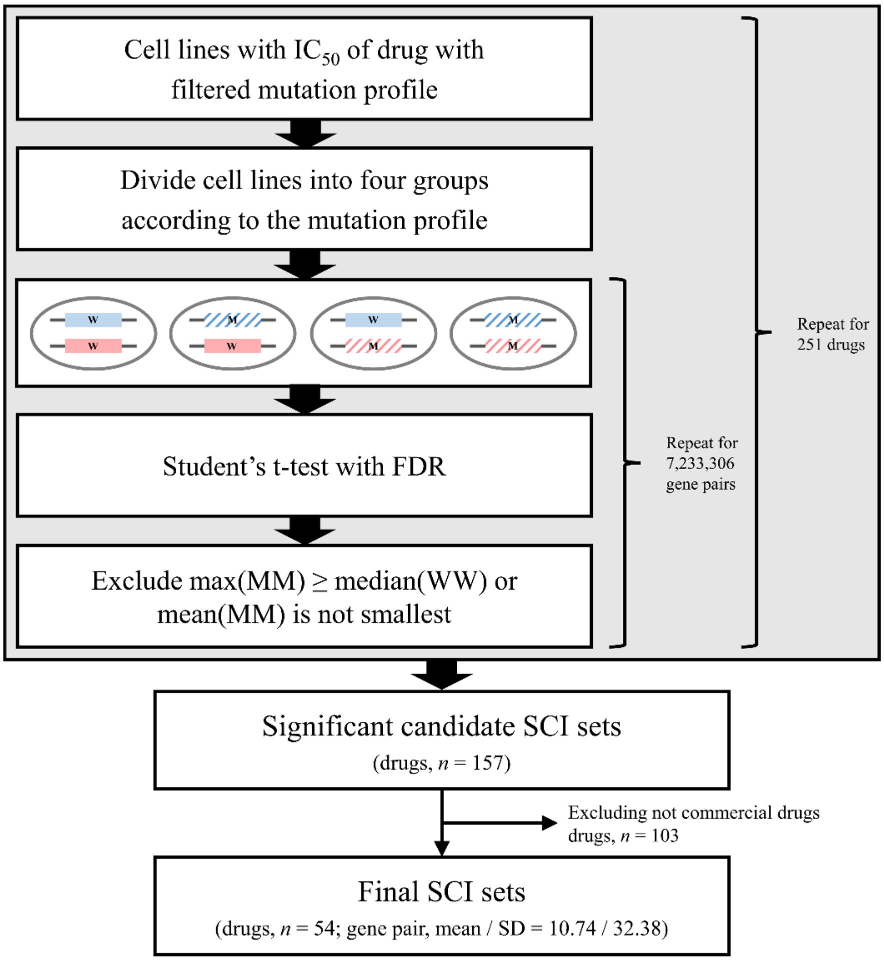

Supplementary Figure S7 | Analysis pipeline to identify candidate SCI sets. To identify gene pairs showing synergistic chemotherapeutic interactions with a specific drug, we compared the IC50 values of the MM group and the other three groups using the Student t-test. Gene pairs with significantly lower IC50 values in MM were further filtered according to the following two criteria: 1) mean IC50 value of MM groups was the smallest among the four groups, and 2) the maximum IC50 value of the MM group was smaller than the median IC50 value of the WW group.

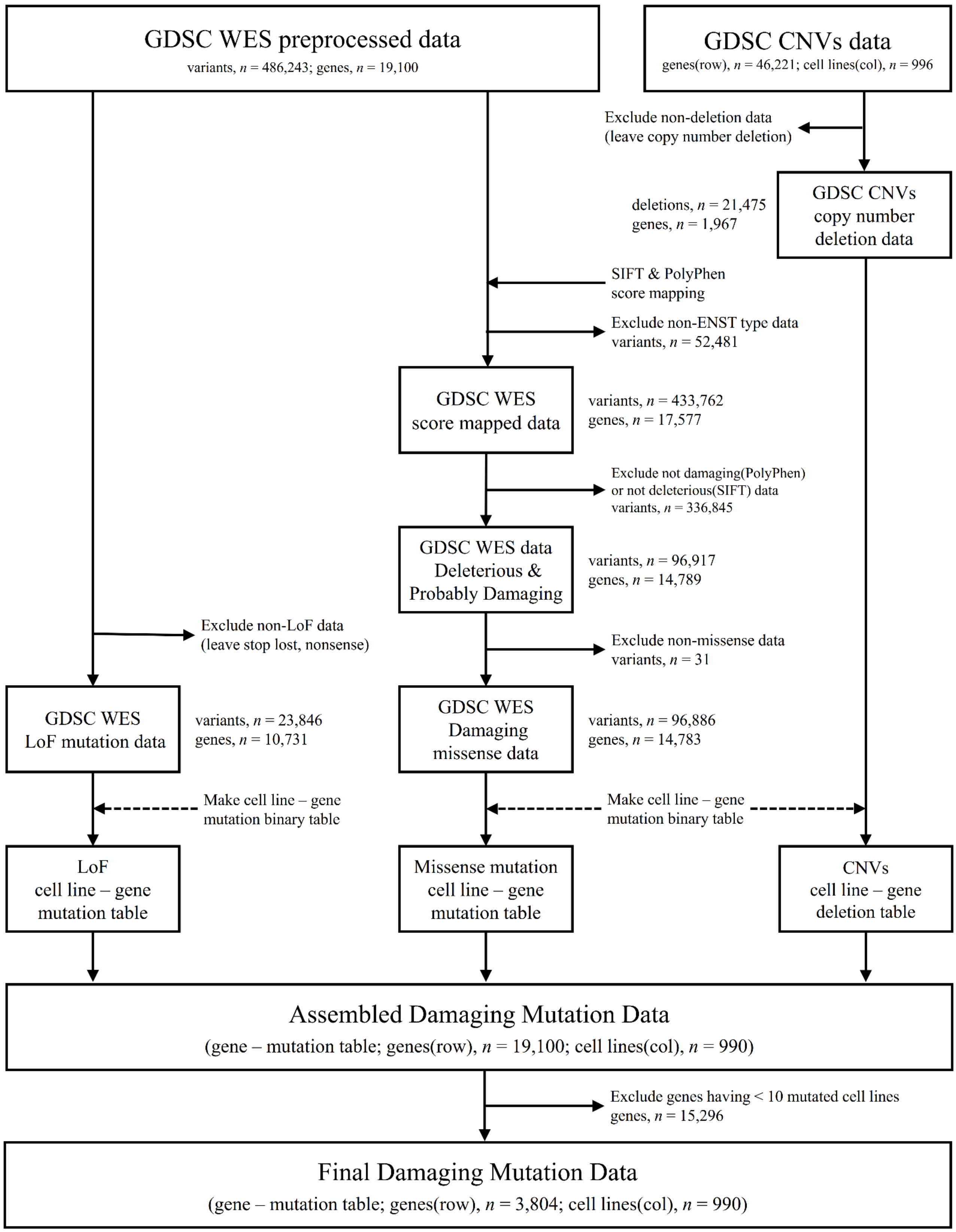

Supplementary Figure S8 | Detailed flowchart of data process for damaging mutation data. GDSC's preprocessed whole exome sequencing (WES) data and copy number variant (CNV) data were used to filter and extract damaging mutation data. Damaging missense mutation, loss of function (LoF) mutation, copy number deletion were assembled for damaging mutation data. SIFT and PolyPhen-2 score mapping was processed at WES data for filtering damaging missense mutation. Then variants were filtered for damaging (PolyPhen-2) and deleterious (SIFT) mutations. Finally, missense mutations were only remaining as damaging missense data. Stop loss and nonsense mutation were defined as LoF mutations. Cell line – gene mutation table were made for all three mutation data groups and merged into single table. After merging, gene filtering based on number of mutated cell lines were processed and final damaging mutation data table was made.

Supplementary Table S1 | Gene-cell line damaging mutation profile binary table (row: cell lines, *n* = 990; column: genes, *n* = 19100). We made the table which contains the mutation profile of 19,100 genes for 990 cell lines. The numbers represent the combined count of mutations including loss of function mutation, damaging missense and copy number deletions.

	A1BG	A1CF	A2M	A2ML1	A4D226	A4GALT	A4GNT	AAAS	AACS	AADAC
201T	0	0	0	0	0	0	0	0	0	0
22RV1	0	0	0	0	0	0	0	0	0	0
23132-87	0	0	0	0	0	0	0	0	0	0
42-MG-BA	0	0	0	0	0	0	0	0	0	0
451Lu	0	0	0	0	0	0	0	0	0	0
5637	0	0	0	0	0	0	0	0	0	0
639-V	0	0	0	1	0	0	0	0	0	0
647-V	0	0	0	0	0	0	0	0	0	0
697	0	0	0	0	0	0	0	0	0	0
769-P	0	0	0	0	0	0	0	0	0	0
786-0	0	0	0	0	0	0	0	0	0	0
8-MG-BA	0	0	0	0	0	0	0	0	0	0
8305C	0	0	0	0	0	0	0	0	0	0
8505C	0	0	0	0	0	0	0	0	0	0
A101D	0	0	0	0	0	0	0	0	0	0
A172	0	0	0	0	0	0	0	0	0	0
A204	0	0	0	0	0	0	0	0	0	0
A2058	0	0	0	0	0	0	0	0	0	0
A253	0	0	0	0	0	0	0	0	0	0
A2780	0	0	0	0	0	0	0	0	0	0
A3-KAW	0	0	0	0	0	0	0	0	0	0
A375	0	0	0	0	0	0	0	0	0	0
A388	0	0	0	0	0	0	0	0	0	0
A4-Fuk	0	0	0	0	0	0	0	0	0	0
A427	0	0	0	0	0	0	0	0	0	0
A431	0	0	0	0	0	0	0	0	0	1

Supplementary Table S2 | Detailed information of final SCI sets. Total 580 SCI sets' detailed information is shown. In this table, name of drug and genes, number of cell lines of separated 4 groups, mean / SD values of IC_{50} for 4 groups, p-value and FDR value is included for each SCI sets.

	A	B	C	D	E	F	G	H	I	J	K	L	M	N	
1	Drug_Name	Gene1_Name	Gene2_Name	WW_N	WM_N	MW_N	MM_N	WW_mean_raw	WM_mean_raw	MW_mean_raw	MM_mean_raw	WW_SD_raw	WM_SD_raw	MW_SD_raw	MI
2	Sunitinib	FAT2	TSPY2	307	65	20	5	2.3847	2.6285	2.5084	1.5201	1.6874	1.7177	1.6532	
3	Sunitinib	FLNB	LRP1	370	14	8	5	2.4317	2.2976	2.9847	0.9906	1.6990	1.2447	1.7357	
4	Paclitaxel	MTAP	WDR87	330	14	49	5	-3.0276	-2.9998	-3.2738	-4.9848	1.9052	2.1241	1.9844	
5	Dasatinib	LGALS9C	OR2T10	307	15	65	7	0.2693	0.3192	0.5213	-2.3293	2.4456	2.3680	2.5875	
6	Dasatinib	MTAP	WDR87	327	14	48	5	0.4806	-0.5710	-0.5675	-3.3742	2.3411	3.0510	2.7681	
7	Dasatinib	DPP10	GYG2P1	207	175	6	6	0.3588	0.2151	1.3378	-2.4807	2.5187	2.4031	2.3601	
8	Bortezomib	ABCB5	LRP1B	344	43	6	5	-5.6884	-5.3831	-5.1139	-7.1935	1.5577	1.5774	1.1168	
9	Bortezomib	KIAA2018	MGAM	369	17	7	5	-5.6512	-5.6706	-5.2681	-7.2720	1.5498	1.6606	1.7299	
10	Bortezomib	LGALS9C	MGAM	306	16	70	6	-5.6607	-5.5019	-5.5714	-7.4549	1.5534	1.5755	1.5531	
11	Bortezomib	APOB	TSPY2	313	67	13	5	-5.6136	-5.8784	-5.2746	-7.0916	1.5470	1.5584	1.7663	
12	Bortezomib	CACNA1H	TBL1Y	204	174	14	6	-5.5760	-5.7849	-4.8804	-7.0905	1.6010	1.4761	1.7031	
13	Seliciclib	PRSS2	ZBTB7A	303	5	80	6	3.9600	4.2310	3.9507	2.9613	1.0235	0.7905	0.8686	
14	Doxorubicin	A2ML1	DNAH8	777	68	11	5	-1.7482	-1.6759	-1.3879	-3.9821	1.6401	1.7903	2.0237	
15	Doxorubicin	LRP1	SHANK2	799	18	39	5	-1.7581	-1.0456	-1.6697	-3.7710	1.6414	2.0453	1.7681	
16	Doxorubicin	MLL2	TUBGCP6	772	13	71	5	-1.7212	-1.4837	-1.9777	-3.8061	1.6573	1.9001	1.6135	
17	Doxorubicin	SHROOM2	TSPY2	656	190	10	5	-1.7545	-1.7246	-1.0525	-3.6694	1.6661	1.6225	1.8363	
18	Etoposide	KIAA1109	STK11IP	820	13	33	5	1.5568	2.0470	2.1988	-0.2094	1.9563	1.8697	1.9760	
19	Etoposide	LRP1B	MITF	760	5	101	5	1.5627	3.1905	1.6902	0.0700	1.9617	2.3834	1.9073	
20	Etoposide	MCM7	RYR3	803	54	8	6	1.5972	1.5511	1.3115	-0.3458	1.9569	2.0196	1.7836	
21	Etoposide	ODZ2	PREX2	810	22	34	5	1.5920	1.0526	1.8471	-0.1585	1.9729	1.3238	1.9356	
22	Etoposide	PKD1L1	TSPY2	653	192	21	5	1.5659	1.6205	1.8928	0.2567	1.9556	1.9744	2.0501	
23	Etoposide	PREX2	TTF2	830	14	22	5	1.6052	1.4311	1.0591	-0.1874	1.9676	2.2305	1.3177	
24	Etoposide	PRSS1	TBL1Y	422	430	14	5	1.5066	1.7174	0.4962	-1.2986	1.9636	1.9211	2.1131	
25	Etoposide	TP53	XIRP1	462	7	394	8	1.4862	2.0851	1.7186	-0.4606	2.0119	1.6845	1.8887	
26	Etoposide	AQR	PREX2	823	21	21	6	1.5985	1.1673	1.7497	-0.3583	1.9740	1.2548	1.8879	
27	Etoposide	CACNA1E	FLNB	810	26	30	5	1.5787	1.1207	2.2589	-0.1972	1.9482	2.2378	1.8799	
28	Etoposide	COL14A1	DOCK2	813	28	25	5	1.5790	1.9230	1.4962	-0.0534	1.9597	1.8273	2.1180	
29	Etoposide	CRB2	RYR3	801	55	10	5	1.5938	1.4786	1.6382	0.0728	1.9567	2.0512	1.8628	
30	Etoposide	CRB2	USH2A	764	92	10	5	1.5622	1.7873	1.6450	0.0591	1.9364	2.1625	1.8555	
31	Etoposide	DNAH7	ITGAX	796	13	56	6	1.5839	1.1616	1.7999	-0.3379	1.9311	2.3751	2.2298	
32	Gemcitabine	AHNAK2	IFNE	730	79	41	6	-2.2992	-2.3650	-2.1331	-4.6901	2.6961	2.9213	2.7678	
33	Gemcitabine	COL4A3	DNAH8	770	66	13	7	-2.2704	-2.4843	-2.5279	-5.1155	2.7326	2.6094	2.2769	
34	Gemcitabine	COL4A3	DNAH9	766	70	15	5	-2.3109	-2.0291	-2.9072	-5.0127	2.7513	2.3828	2.3394	
35	Gemcitabine	COL4A3	RELN	788	48	15	5	-2.2721	-2.5356	-2.7643	-5.4411	2.7112	2.9156	2.1825	

Supplementary Table S3 | Brief information of candidate SCS interaction gene pairs, genes and cell lines of each 54 drugs. Mean, standard deviation, minimum and maximum value of gene pairs, genes and cell lines per drug of final SCS interaction sets are shown.

	**Mean / SD**	**Min**	**Max**
Gene pairs per drug (n = 538)	10.74 / 32.38	1	234
Genes per drug (n = 456)	14.67 / 33.18	2	232
MM cell lines per drug (n = 471)	25.81 / 34.64	5	205

Supplementary Table S4 | Number of patients according to the mutational profile of *BRAF* related synergistic interaction sets and status of BRAF/MAPK inhibitor treatment.

	BRAF inhibitor				MEK inhibitor		RAF inhibitor
	**dabrafenib**	**sorafenib (Nexavar)**	**vemurafenib**	**Unknown***	**selumetinib (AZD6244)**	**trametinib**	**regorafenib**
**WW‡ (*n* = 30)**	-	28 (93.3%)	-	-	1 (3.3%)	-	1 (3.3%)
**WM†‡ (*n* = 22)**	1 (4.5%)	20 (90.9%)	1 (4.5%)	-	1 (4.5%)	-	1 (4.5%)
**MW‡ (*n* = 2)**	-	-	1 (50.0%)	1 (50.0%)	-	-	-
**MM†‡ (*n* = 11)**	4 (36.4%)	-	9 (81.8%)	1 (9.1%)	-	2 (18.2%)	-
**Total (*n* = 65)**	5 (7.7%)	48 (73.8%)	11 (16.9%)	2 (3.1%)	2 (3.1%)	2 (3.1%)	2 (3.1%)

† In MM group, four patients used several drugs. In WM group, two patients used several drugs.

‡ Patients belong to MM and MW group were all skin cutaneous melanoma. In WM group, there were eight types of cancers (hepatocellular carcinoma, 13 cases; kidney cancer including papillary, clear cell and kidney chromophobe, 5 cases; and one case for each adrenocortical carcinoma, lung squamous cell carcinoma, sarcoma, skin cutaneous melanoma). In WW group, there were nine types of cancers (liver hepatocellular carcinoma, 15 cases; kidney cancer including papillary, clear cell and kidney chromophobe, 10 cases; and one case for each brain lower grade glioma, colon adenocarcinoma, sarcoma, glioblastoma multiforme, uterine corpus endometrial carcinoma). MM represent both *BRAF* and multi-drug combined partner genes mutated patient group. MW represent BRAF mutated and wild type partner genes. WM represent wild type BRAF and mutated type partner genes. WW represent both BRAF and partner genes wild type.

* Described as a BRAF inhibitor or GSK BRAF inhibitor.

Supplementary Table S5 | Melanoma-specific comparison for the nine partner genes which driven by previous analysis

Drug_Name	Gene1_Name	Gene2_Name	Number of cell lines				P values (Student's t-test)		
			WW	WM	MW	MM	WW vs MM	WM vs MM	MW vs MM
Dabrafenib	*BRAF*	*C10orf112*	8	2	30	5	0	0.00004	0.0223
Dabrafenib	*BRAF*	*COL2A1*	9	1	30	5	0.00002		0.4543
Dabrafenib	*BRAF*	*COL4A4*	9	1	31	4	0		0.05777
Dabrafenib	*BRAF*	*GPR112*	10	0	30	5	0		0.02758
Dabrafenib	*BRAF*	*NBEA*	8	2	30	5	0	0.00078	0.03071
Dabrafenib	*BRAF*	*NLRP3*	10	0	32	3	0.00489		0.7809
Dabrafenib	*BRAF*	*TTN*	9	1	28	7	0		0.00165
Dabrafenib	*BRAF*	*ZNF234*	8	2	33	2	0.00003	0.03247	0.02484
Refametinib	*BRAF*	*CSMD1*	9	3	26	11	0.2431	0.5486	0.1675
Refametinib	*BRAF*	*LCE3C*	8	4	26	11	0.3237	0.4166	0.1945
Refametinib	*BRAF*	*NBEA*	10	2	32	5	0.00481	0.5387	0.00071
Trametinib	*BRAF*	*C10orf112*	10	2	31	5	0.1175	0.9687	0.107
Trametinib	*BRAF*	*COL2A1*	11	1	31	5	0.4947		0.3163
Trametinib	*BRAF*	*CSMD1*	9	3	26	10	0.2554	0.3411	0.06443
Trametinib	*BRAF*	*GPR112*	12	0	31	5	0.01335		0.00347
Trametinib	*BRAF*	*NBEA*	10	2	31	5	0.02787	0.3044	0.00505
Trametinib	*BRAF*	*PLG*	9	3	34	2	0.1319	0.323	0.1416

Supplementary Table S6 | The number of cell lines in each SCS group according to the mutational status of the genes related to *BRAF* inhibitor resistance and *BRAF* amplifications.

GDSC cell lines	Mutational status of SCS groups of the known genes related to *BRAF* inhibitor resistance including *BRAF* amplification														
	MAP2K1 (MEK1)	***MAP2K2 (MEK2)***	***MAP2K3***	***MAP2K4***	***MAP2K5***	***MAP2K6***	***MAP2K7***	***PIK3CA***	***PTEN***	***RAC1***	***CDK4***	***AKT1***	***NF1***	***NRAS***	***BRAF* amplification**	**Total**
**WW*** **(*n* = 405)**	1 (0.2%)	-	3 (0.7%)	4 (1.0%)	2 (0.4%)	-	5 (1.2%)	15 (3.7%)	19 (4.7%)	4 (1.0%)	1 (0.2%)	1 (0.2%)	11 (2.7%)	-	1 (0.2%)	62 (15.3%)
**WM (*n* = 461)**	6 (1.3%)	4 (0.9%)	4 (0.9%)	10 (2.2%)	3 (0.7%)	1 (0.2%)	10 (2.2%)	32 (6.9%)	38 (8.2%)	3 (0.7%)	1 (0.2%)	7 (1.5%)	25 (5.4%)	2 (0.4%)	2 (0.4%)	115 (24.9%)
**MW (*n* = 23)**	-	-	-	2 (8.7%)	-	-	-	2 (8.7%)	-	-	-	1 (4.3%)	-	-	-	5 (21.7%)
**MM (*n* = 51)**	-	1 (2.0%)	-	4 (7.8%)	-	1 (2.0%)	-	2 (3.9%)	3 (5.9%)	-	1 (2.0%)	-	2 (3.9%)	-	1 (2.0%)	12 (23.5%)
**Total† (*n* = 940)**	7 (0.7%)	5 (0.5%)	7 (0.7%)	20 (2.1%)	5 (0.5%)	2 (0.2%)	15 (1.6%)	51 (5.4%)	60 (6.4%)	7 (0.7%)	3 (0.3%)	9 (1.0%)	38 (4.0%)	2 (0.2%)	4* (0.4%)	194 (20.6%)

We compared the mutational profile of well-known resistance genes including *BRAF* amplification between the *BRAF*-related SCS groups that we identified from the previous analysis in the GDSC database. In this comparison, the partner genes were eleven genes derived from the clustering analysis including all three BRAF-inhibitors.

* The front capital character denotes the *BRAF* gene and the latter of the two capital characters denotes the mutational status of the multi-drug combined partner genes.

† Only cell lines tested with any of dabrafenib, refametinib, and trametinib are included and 936 cell lines were able to check CNV data.

SCS: synergistic chemo-sensitivity

Supplementary Table S7| The number of patients in each SCS group according to the mutational status of genes related to *BRAF* inhibitor resistance and *BRAF* amplifications from the TCGA data.

**TCGA**	**Known mutations/CNVs related to *BRAF* inhibitor resistance including *BRAF* amplification**											
	MAP2K1	***MAP2K2***	***MAP2K3***	***PIK3CA***	***PTEN***	***RAC1***	***CDK4***	***AKT1***	***NF1***	***NRAS***	***BRAF* amplification**	**Total**
**WW*** (***n* = 30**)	-	-	1 (3.3%)	1 (3.3%)	1 (3.3%)	-	-	-	1 (3.3%)	1 (3.3%)	-	4 (13.3%)
**WM** (***n* = 22**)	-	1 (4.5%)	-	-	-	-	-	-	3 (13.6%)	-	-	4 (18.2%)
**MW** (***n* = 2**)	-	-	-	-	-	-	1 (50.0%)	-	-	-	-	1 (50.0%)
**MM** (***n* = 11**)	1 (9.1%)	-	1 (9.1%)	-	1 (9.1%)	-	1 (9.1%)	-	1 (9.1%)	-	1 (9.1%)	6 (54.5%)
**Total** (***n* = 65**)	1 (1.5%)	1 (1.5%)	2 (3.1%)	1 (1.5%)	2 (3.1%)	-	2 (3.1%)	-	5 (7.7%)	1 (1.5%)	1* (1.5%)	15 (23.1%)

We compared the mutational profile of well-known resistance genes including *BRAF* amplification between the *BRAF*-related SCS groups' patients in the TCGA database. In this comparison, the partner genes were eleven genes derived from the clustering analysis including all three BRAF-inhibitors from the previously derived from the analysis of GDSC. Only 62 patients were able to check CNV data.

* The front capital character denotes the *BRAF* gene and the latter of the two capital characters denotes the mutational status of the multi-drug combined partner genes.

SCS: synergistic chemo-sensitivity

Supplementary Table S8 | The number of patients in each SCS according to the mutational status of genes related to *BRAF* inhibitor resistance and *BRAF* amplifications from the TCGA data.

TCGA Non-*TTN*	Known mutations/CNVs related to *BRAF* inhibitor resistance including *BRAF* amplification											
	MAP2K1	***MAP2K2***	***MAP2K3***	***PIK3CA***	***PTEN***	***RAC1***	***CDK4***	***AKT1***	***NF1***	***NRAS***	***BRAF* amplification**	**Total**
**WW*** (***n* = 40)**	-	-	1 (2.5%)	1 (2.5%)	1 (2.5%)	-	-	-	2 (5.0%)	1 (2.5%)	-	5 (12.5%)
**WM** (***n* = 12)**	-	1 (8.3%)	-	-	-	-	-	-	2 (16.7%)	-	-	3 (25.0%)
**MW** (***n* = 8)**	-	-	-	-	1 (12.5%)	-	2 (25.0%)	-	1 (12.5%)	-	-	4 (50.0%)
**MM** (***n* = 5)**	1 (20.0%)	-	1 (20.0%)	-	-	-	-	-	-	-	1 (20.0%)	3 (60.0%)
**Total** (***n* = 65)**	1 (1.5%)	1 (1.5%)	2 (3.1%)	1 (1.5%)	2 (3.1%)	-	2 (3.1%)	-	5 (7.7%)	1 (1.5%)	1* (1.5%)	15 (23.1%)

We compared the mutational profile of well-known resistance genes including *BRAF* amplification between the *BRAF*-related SCS groups' patients in the TCGA database. In this comparison, the partner genes were ten genes except *TTN* derived from the clustering analysis including all three BRAF-inhibitors from the previously derived from the analysis of GDSC. Only 62 patients were able to check CNV data.

* The front capital character denotes the *BRAF* gene and the latter of the two capital characters denotes the mutational status of the multi-drug combined partner genes.

SCS: synergistic chemo-sensitivity

Supplementary Table S9 | Detailed information for 49 *BRAF* related MM group cell lines. 49 *BRAF* related MM group cell lines' detailed information is shown. In this table, name of cell lines and its origins, mutation counts of LoF, CNV deletion, damaging missense for *BRAF* CPs genes and all genes, whether cell lines' IC50 were measured by three drugs, whether cell lines are included in MM cell lines of SCI sets, whether these cell lines are *BRAF* mutated, and whether these cell lines are included in MM cell lines of *BRAF* related SCI sets are checkable.

Name	Origin_1	Origin_2	LOF_BRAF_CPs	CNV_BRAF_CPs	Missense_BRAF_CPs	LOF_all	CNV_all	Missense_all	Dabrafenib_drug_test	Refametinib_drug_test
451Lu	skin	melanoma	0	0	2	26	22	99	TRUE	TRUE
A101D	skin	melanoma	0	1	2	9	23	30	TRUE	TRUE
A375	skin	melanoma	1	0	2	23	24	83	TRUE	TRUE
BHT-101	thyroid	thyroid	0	1	1	3	23	30	TRUE	TRUE
C32	skin	melanoma	2	0	1	14	5	47	TRUE	TRUE
CAL-12T	lung_NSCLC	lung_NSCLC_not specified	0	1	1	35	22	133	TRUE	TRUE
CL-34	large_intestine	large_intestine	0	1	1	44	23	223	FALSE	TRUE
COLO-679	skin	melanoma	1	0	3	31	20	109	TRUE	TRUE
COLO-783	skin	melanoma	2	0	3	23	0	100	TRUE	TRUE
COLO-792	skin	melanoma	2	0	2	104	2	378	TRUE	TRUE
CP50-MEL-B	skin	melanoma	0	1	1	7	4	29	TRUE	TRUE
DU-145	urogenital_system	prostate	3	0	5	132	5	557	TRUE	TRUE
ES-2	urogenital_system	ovary	0	0	3	24	22	105	TRUE	TRUE
G-361	skin	melanoma	0	0	3	13	24	47	TRUE	TRUE
G-MEL	skin	melanoma	2	0	4	34	7	90	TRUE	TRUE
GCT	soft_tissue	soft_tissue_other	1	0	2	37	21	135	TRUE	TRUE
HCT-15	large_intestine	large_intestine	2	1	11	296	2	1274	TRUE	TRUE
Hey	urogenital_system	ovary	0	1	1	7	26	39	TRUE	TRUE
HT-144	skin	melanoma	0	1	2	7	17	41	TRUE	TRUE
HT-29	large_intestine	large_intestine	1	1	1	27	22	56	TRUE	TRUE
HT55	large_intestine	large_intestine	0	1	2	72	26	243	FALSE	TRUE
HTC-C3	thyroid	thyroid	0	1	2	12	21	59	TRUE	TRUE
IGR-1	skin	melanoma	1	1	3	67	9	203	FALSE	TRUE
IGR-37	skin	melanoma	2	0	1	14	18	64	TRUE	TRUE
K2	skin	melanoma	1	0	2	41	25	141	TRUE	TRUE
K5	thyroid	thyroid	0	1	2	8	11	40	TRUE	TRUE
LOXIMVI	skin	melanoma	0	0	3	17	24	102	TRUE	TRUE
LS-180	large_intestine	large_intestine	3	0	1	59	24	338	TRUE	TRUE
LS-411N	large_intestine	large_intestine	2	1	6	158	20	641	TRUE	TRUE
LS-513	large_intestine	large_intestine	2	1	2	16	5	52	TRUE	TRUE
M14	skin	melanoma	3	1	5	59	28	237	TRUE	TRUE
MDST8	large_intestine	large_intestine	0	0	5	47	21	159	TRUE	TRUE
MEL-HO	skin	melanoma	0	0	2	9	19	44	TRUE	TRUE
